# Chemokines in Prediabetes and Type 2 Diabetes: A Meta-Analysis

**DOI:** 10.3389/fimmu.2021.622438

**Published:** 2021-05-13

**Authors:** Xiongfeng Pan, Atipatsa C. Kaminga, Shi Wu Wen, Aizhong Liu

**Affiliations:** ^1^ Department of Epidemiology and Health Statistics, Xiangya School of Public Health, Central South University, Changsha, China; ^2^ Hunan Provincial Key Laboratory of Clinical Epidemiology, Xiangya School of Public Health, Central South University, Changsha, China; ^3^ Department of Mathematics and Statistics, Mzuzu University, Mzuzu, Malawi; ^4^ OMNI Research Group, Ottawa Hospital Research Institute, Ottawa, ON, Canada; ^5^ Department of Obstetrics and Gynaecology and School of Epidemiology and Public Health, University of Ottawa Faculty of Medicine, Ottawa, ON, Canada

**Keywords:** chemokines, type 2 diabetes, prediabetes, inflammation, meta-analysis

## Abstract

**Background:**

A growing number of studies found inconsistent results on the role of chemokines in the progression of type 2 diabetes (T2DM) and prediabetes (PDM). The purpose of this meta-analysis was to summarize the results of previous studies on the association between the chemokines system and T2DM/PDM.

**Methods:**

We searched in the databases, PubMed, Web of Science, Embase and Cochrane Library, for eligible studies published not later than March 1, 2020. Data extraction was performed independently by 2 reviewers, on a standardized, prepiloted form. Group differences in chemokines concentrations were summarized using the standardized mean difference (SMD) with a 95% confidence interval (CI), calculated by performing a meta-analysis using the random-effects model.

**Results:**

We identified 98 relevant studies that investigated the association between 32 different chemokines and T2DM/PDM. Altogether, these studies involved 14,708 patients and 14,574 controls. Results showed that the concentrations of CCL1, CCL2, CCL4, CCL5, CCL11, CXCL8, CXCL10 and CX3CL1 in the T2DM patients were significantly higher than that in the controls, while no difference in these concentrations was found between the PDM patients and controls.

**Conclusion:**

Progression of T2DM may be associated with elevated concentrations of chemokines.

**Meta-Analysis Registration:**

PROSPERO, identifier CRD42019148305.

## Background

With the growing global prevalence of obesity, the incidence of type 2 diabetes (T2DM) continues to increase and this has become one of the most important public health problems worldwide (1). T2DM cannot be completely cured, which seriously affects the quality of life of patients (2). Currently, research has shown that targeted anti-inflammatory drugs have great potential in improving insulin sensitivity and B-cell function (3). Meanwhile, a newly published meta-analysis showed that T2DM risk was closely related to the increased levels of inflammatory cytokines such as Tumor necrosis factor-α (TNF-α), Interleukin-1 beta (IL-1β), Interleukin-6 (IL-6), Interleukin-18 (IL-18), and C-reactive protein (CRP) (4). Also, accumulating evidence support an important hypothesis that there may be a complex interaction between chronic inflammation and the progression of diabetes (5, 6).

Furthermore, studies have shown that in addition to these inflammatory markers, chemokines have become key signaling molecules regulating the pathophysiological process of T2DM (7). Chemokines are the largest family of cytokines composed of four subfamilies (C, CC, CXC, and CX3C) (8). Thus, chemokines have been shown to function as signaling molecules in the inflammatory response, which can activate various pro-inflammatory mediators and induce various inflammatory factors (8, 9). These inflammatory factors stimulate the activation of cytokine signaling proteins, which eventually block the activation of insulin signaling receptors in pancreatic cells, and further induce insulin resistance (IR); hence participate in the progression of prediabetes (PDM) and T2DM (10).

The names of chemokines are not uniform at present. For example, there are about 50 chemokines acting on 23 discrete receptors, and studies on different chemokines in PDM and T2DM patients often produce inconsistent results (11, 12). It is unclear which chemokines vary in concentrations in PDM and T2DM patients to be considered as potential new biomarkers.

So far, there has not been a systematic review/meta-analysis on the role of chemokines in the progression of PDM and T2DM. Noteworthy, it is difficult to obtain statistically stable results from a single study. Thus, meta-analysis provides a gold standard method for aggregating data from several similar studies, and helps to improve statistical precision to achieve stable results. Therefore, the purpose of this study was to compare the chemokines concentrations between patients with PDM or T2DM and the control group by means of a meta-analysis, using as many variants of historical and current chemokines as possible retrieved through a wide range of search terms. The illustration of signal transduction pathway of chemokines in the pathophysiological process of PDM and T2DM could provide new targets and strategies for the prevention, diagnosis, and treatment of PDM and T2DM.

## Methods

### Search Strategy and Selection Criteria

The protocol of this systematic review has been registered in PROSPERO database (https://www.crd.york.ac.uk/PROSPERO), CRD42019148305. Also, the study followed the Preferred Reporting Items for Systematic Reviews and Meta-Analyses (PRISMA) guidelines and the Cochrane Handbook (13).

Due to the inconsistent naming format of chemokines in published articles, the search strategy involved as many variant nomenclatures of chemokines as possible. According to the purpose of this study, two independent reviewers (AL and AK) selected relevant articles. In this regard, the electronic databases of Embase, Web of Science, Cochrane Library, and PubMed were searched for relevant articles published not later than March 1, 2020. The complete search strategy is shown in **Appendix 1**. Truncation, wildcards and boolean operators were used to allow for the retrieval of as many variant nomenclatures of chemokines as possible. Experienced librarians designed the search algorithm and customized it to meet the search requirements of each of the preceding electronic databases.

The following were inclusion criteria for eligible studies (1): the study design was longitudinal, cross-sectional or case-control; (2) the study reported the method for diagnosing PDM or T2DM; and (3) the study reported a mean chemokine concentration and its standard deviation (SD), or these could be obtained from the corresponding author. The exclusion criteria were: (1) letters, reviews, case reports, comments and non-human studies; (2) studies reported PDM or T2DM in combination with other diseases; (3) PDM and T2DM patients had the chemokines pharmacologically challenged before their measurements.

### Data Extraction

Two independent reviewers (SW and AK) removed the duplicates using EndNote (version X9.1) and entered the eligible studies into a database constructed by EpiData (version 3.0) (14, 15). In this database, customized data extraction Excel spreadsheets were used to extract the data for this study (16). Differences between the two independent reviewers were resolved by involving a third reviewer (AL). Therefore, the following data were extracted from the eligible studies:(1) the first author’s last name and year of publication; (2) the country where the research was conducted; (3) type of control group; (4) PDM and T2DM patients characteristics such as duration of diabetes (years), waist circumference, waist to hip ratio, systolic blood pressure (SBP), diastolic blood pressure (DBP), fasting plasma glucose/fasting blood glucose (FPG), 2h postprandial blood glucose, Body Mass Index (BMI), age and gender of patients; (5) chemokine sample detection method and storage temperature; (6) sample material of chemokine, mean and standard deviation of chemokine concentration, blood biochemical indexes and other sample characteristics. Finally, the Newcastle-Ottawa Quality Assessment Scale (NOS) was used to assess the risk of bias and quality of eligible studies (17).

### Statistical Analysis

All statistical analyses were performed using the package meta in R (version 3.5.0) (18). Noteworthy, different chemokine assessment methods were used for different studies. Therefore, to be conservative, the random-effects model was used to determine the standardized mean difference (SMD) and 95% confidence interval (CI) for continuous data (19). Using the restricted maximum-likelihood estimator, the SMD was calculated using the Cohen’s d method to synthesize the effect size across studies (20, 21). Furthermore, heterogeneity among studies was assessed by the Cochran’s Q-test and quantified by the *I*² statistic (22, 23). Thus, *I²=*100% indicated maximal heterogeneity and *I²=*0% indicated no heterogeneity (23). The preceding analysis was first applied on all chemokines concentrations, then on each specific type of chemokine. Moreover, subgroup analysis was conducted to explore whether gender (Male and Female subgroups), age (≤60 and >60 subgroups), and method for measuring chemokines (Luminex method and ELISA method subgroups) had any influence on the results. Sensitivity analysis was carried out using the leave-one-out method. When the number of studies reporting the main results was 10 or more, publication bias was assessed using the funnel plot and Egger’s test (24). The significance level was defined as p<0.05 in all analyses, and all tests were two-sided.

## Results

### Literature Search

The search strategy retrieved a total of 8,775 studies, of which 2,720 were from PubMed, 3,780 from Embase, 1,386 from Web of Science, and 889 from Cochrane Library. Thus, 7,922 articles were excluded for being unrelated to this study. Then the full texts of the 853 articles were assessed, and it was found that 755 articles were not eligible for this study, hence they were excluded. Finally, 98 articles were included in the meta-analysis. **Appendix 1** shows a flow diagram presenting the selection process of the included studies.

### Characteristics of Eligible Studies

**Figure 1:**
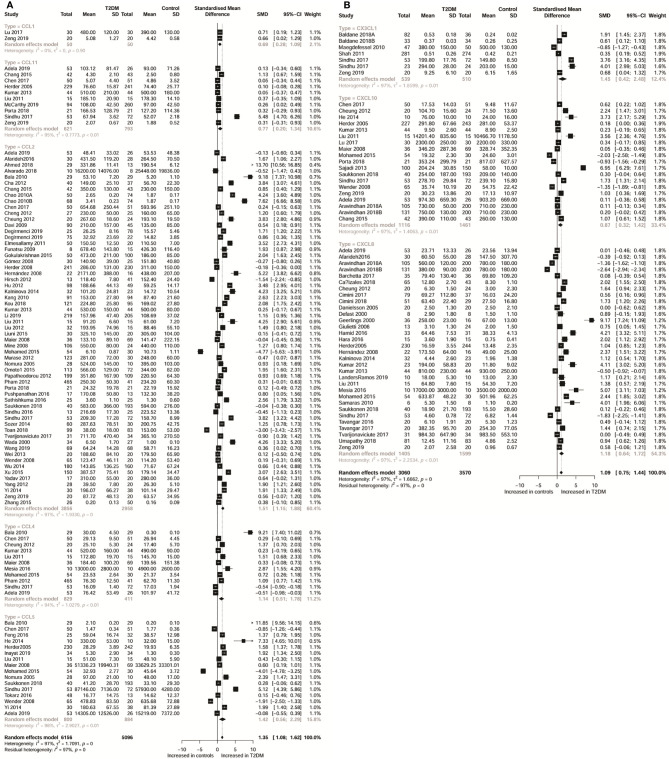
Forest plot of CC chemokine **(A)** and CXC chemokine **(B)** between T2DM patients and controls. Study effect sizes of chemokines differences between T2DM and controls. Each data marker represents a study, and the size of the data marker is proportional to the total number of individuals in that study. The summary effect size for each chemokines is denoted by a diamond. T2DM, type 2 diabetes mellitus; SMD, standardized mean difference.

**Figure 2:**
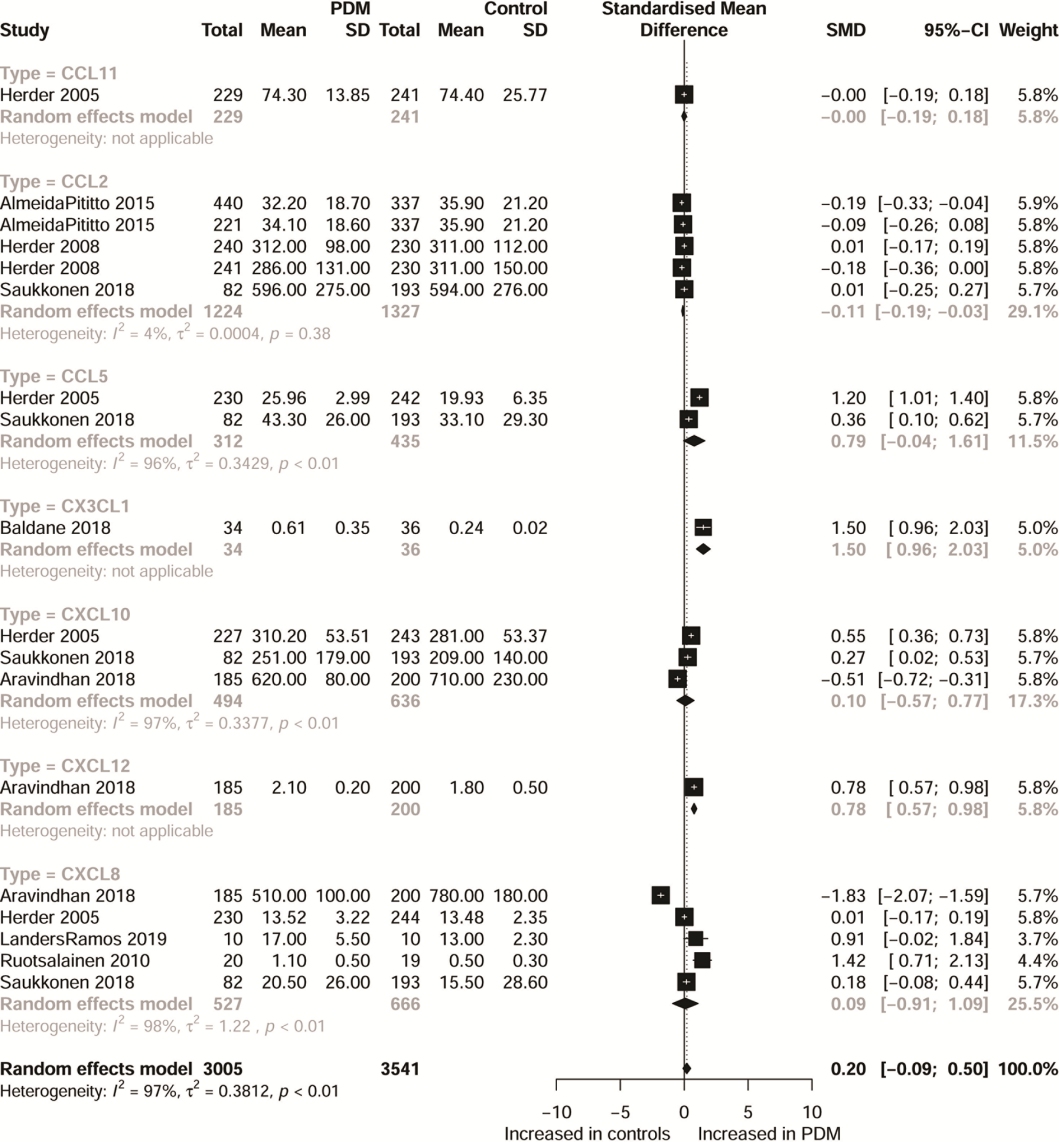
Forest plot of chemokine between PDM patients and controls. Study effect sizes of chemokines differences between PDM and controls. Each data marker represents a study, and the size of the data marker is proportional to the total number of individuals in that study. The summary effect size for each chemokines is denoted by a diamond. PDM, prediabetes diabetes mellitus; SMD, standardized mean difference.

Characteristics of the included studies are shown in **Table 1** and **Appendix 2**. Moreover, **Appendix 3** shows a systematic summary of the classification of chemokines and the distribution of the cell type of chemokine receptors such as neutrophils, monocytes, mast cells, basophils, dendric cells, CD8 T cells, and natural killer (NK) cells, in the immune microenvironment. As regards T2DM, the eligible articles included 19 different CC chemokines comparisons involving 7,558 patients and 6,140 controls (**Figure 1** and **Appendix 4** show forest plots for these chemokines). Also, the eligible articles included a comparison of 13 different CXC chemokines involving 4,145 patients and 4,893 controls (**Figure 1** and **Appendix 5** show forest plots for these chemokines). For PDM, the eligible articles included 7 different chemokines comparisons involving 3,005 patients and 3,541 controls (**Figure 2** and **Appendix 6** show forest plots for these chemokines).

**Table 1:** Characteristics of included studies.

Study		Material	Country	NOS	Male gender n(%)	BMI	Mean Age
Adela 2019	(25)	Serum	India	7	28(52.8%)	26.5±23.5	46.5±8.5
Afarideh 2016	(26)	Venous blood	Iran	8	17(56.6%)	26.4	54.5
Ahmed 2018	(27)	Serum	Egypt	7	0(0.0%)	29.50±0.05	50.48±1.38
Alicka 2019	(28)	Subcutaneous adipose tissues	Poland	6	6(50.5%)	41.50±5.50	36–69
AlmeidaPititto 2015	(29)	Blood	Brazil	7	NR	28.1 (4.2)	46.8 (4.6)
Alvarado 2018	(30)	Blood	Sweden	6	6(58.3%)	NR	56.3±11.9
Aravindhan 2018	(31)	Serum	India	8	69(65.7%)	27±4	46±9
Bala 2010	(32)	Serum	Germany	8	18(62.1%)	31.0±2.3	61.0±2.3
Baldane 2018A	(33)	Venous blood	Turkey	8	36(43.9%)	31.1±5.4	53.6±9.7
Baldane 2018B	(34)	Blood	Turkey	7	16(48.4%)	27.99±2.31	50.0±10.2
Barchetta 2017	(35)	Blood	Italy	8	47(66.2%)	26.6±1.8	51.9±9.2
Cañizales 2018	(36)	Plasma	Mexico	8	40(61.5%)	30.04±5.98	50.03±7.67
Capone 2015	(37)	Blood	Italy	5	11(64.7%)	24.4±0.8	61.8±5.2
Cha 2012	(38)	Venous blood	Korea	8	55(47.4%)	25.3±3.11	45.8±13.8
Chang 2015	(39)	Fasting plasma	China	6	22 (52.4%)	26.71±4.77	57.0±2.0
Chao 2010	(40)	Peripheral blood	China	8	57(48.3%)	25.9±4.8	57.6±10.2
Chen 2017	(41)	Aqueous humor	China	6	24(51.0%)	22.6±2.4	58.8±8.9
Cheng 2012	(42)	Serum	China	8	37(67.3%)	25.56±2.47	68.11±7.53
Cheung 2012	(43)	Aqueous humor	Singapore	6	18(66.7%)	NR	67.4±10.7
Cimini 2017	(44)	Fasting blood	Italy	7	45(56.9%)	33.65±6.3	58±9
Cimini 2018	(45)	Fasting blood	Italy	7	32(62.8%)	29.1±6.5	58±11
Danielsson 2005	(46)	Blood	Sweden	5	20(100.0%)	NR	74±3
Davi 2009	(47)	Blood	Italy	6	51(56.6%)	30.7±1.7	72±10
Defast 2000	(48)	Blood	Canada	5	4(50.0%)	29±3	61±5
Degirmenci 2019	(49)	Serum	Turkey	6	36(48.0%)	33.11±5.29	60.04±7.72
Derakhshan 2012	(50)	Peripheral blood	Iran	6	82(41.0%)	NR	40±9
Elmesallamy 2011	(51)	Plasma	Greece	5	13(43.3%)	20.65±0.95	11.47±0.66
Feng 2016	(52)	Blood	China	7	19(76.0%)	26.46±4.60	53.76±8.89
Funatsu 2009	(53)	Vitreous fluid	Japan	6	25(47.2%)	NR	61.2±7.2
Geerlings 2000	(54)	Blood	the Netherlands	6	0(0.0%)	NR	45±4
Giulietti 2006	(55)	Blood	Belgium	6	6(46.15%)	31.3±4.5	62±4
Gokulakrishnan 2015	(56)	Serum	India	7	25(50.0%)	22.3±2.0	20.1±3.2
Gómez 2008	(57)	Blood	Spain	8	12(40.0%)	28.6±2.5	48.5±5.8
Gong 2016	(58)	Serum	China	6	35(60.3%)	23.86±2.26	61.16±14.38
Hamid 2016	(59)	Blood	Pakistan	7	17(51.5%)	26.00±3.70	64.18±3.31
Hara 2016	(60)	Blood	Brazil	5	0(0.0%)	31.66±6.96	32.55±5.94
He 2014	(61)	Venous peripheral blood	China	5	6(60.0%)	28.0±6.7	32±7
Herder 2005	(62)	Blood	Germany	8	137(58.0%)	30.9±4.5	65.1±5.1
Herder 2008	(63)	Blood	Germany	8	137(58.0%)	30.9±4.5	65.1±5.1
Hernández 2008	(64)	Vitreous fluid	Spain	5	9(40.9%)	NR	68±5
Hirsch 2012	(65)	Blood	Brazil	6	7(15.3%)	44.8±7.8	55.7±1.2
Hu 2012	(66)	Blood	China	7	21(47.7%)	24.02±3.46	52.56±9.40
Huang 2012	(67)	Serum	China	5	108(54.0%)	NR	56.7±3.0
Inayat 2019	(68)	Blood	Pakistan	6	18(52.9%)	28±19	46±3
Kalninova 2014	(69)	Plasma	Slovakia	7	NR	28.9±7.3	63.00±8.50
Kang 2010	(70)	Plasma	South Korea	8	41(45.0%)	25.5±3.21	53.8±10.8
Kou 2018	(71)	Blood	China	7	51 (54.8%)	25.47±3.32	60.15±12.32
Kumar 2012	(72)	Pancreata	India	6	13(56.5%)	NR	38.1±9.2
Kumar 2013	(73)	Plasma	India	7	31(70.5%)	23.90±9.56	45±3
LandersRamos 2019	(74)	Aqueous humor	Korea	5	30(46.9%)	NR	56.81±7.96
Lareyre 2018	(75)	Plasma	France	5	NR	25.8±4.4	73±5
Li 2019	(76)	Blood	China	8	108(49.31%)	25.04±2.47	44.64±8.53
Liu 2011	(77)	Tears	China	6	8(53.3%)	NR	61.07±2.16
Liu 2012	(78)	Blood	China	7	21 (65.6%)	24.37±3.93	51.97±16.57
Liuni 2015	(79)	Blood	India	6	9(30.0%)	27.5±4.03	73.4±11.2
Lu 2017	(80)	Peripheral blood mononuclear cell、Heparinized venous blood、plasma	China	6	22(73.3%)	NR	54.20±3.86
Maegdefessel 2010	(81)	Blood	Germany	5	37(78.0%)	28.2±5	64.0±9
Maier 2008	(82)	Serum	Australia	5	15(41.6%)	NR	66.2±12.2
Mangialardi 2019	(83)	Bone marrow	UK	5	7(50.0%)	33±2	67±3
McCarthy 2019	(84)	Blood	USA	5	70(74.5%)	NR	68±11.3
Mesia 2016	(85)	Peripheral blood	USA	5	6(60.0%)	36.4±10.95	61.3±5.4
Mine 2008	(86)	Peripheral blood	Japan	6	55(51.9%)	23.7±3.4	65.2±9.6
Mohamed 2015	(87)	Blood	Norway	5	7(29.2%)	NR	50.79±2.05
Murase 2012	(88)	Blood	Japan	6	78 (63.4%)	24.3±4.2	62.9±7.8
Nomura 2005	(89)	Blood	Japan	7	12(42.9%)	23.7±3.8	65±11
Omoto 2015	(90)	Blood	Japan	6	60(53.1%)	26.1±3.9	62±6
Papatheodorou 2012	(91)	Blood	Greece	8	96(48.2%)	31.24±5.1	65.6±9.2
Pham 2012	(92)	Serum	Germany	5	263(56.5%)	30.3±7.0	56.3±8.1
Porta 2018	(93)	Blood	Italy	6	11(52.3%)	27.00±4.00	63.00±9.00
Prechel 2018	(94)	Blood specimens	USA	5	20 (40.0%)	35.1	57.52
Pushpanathan 2016	(95)	Blood	India	5	NR	30.3±5.16	51.52±13.69
Ruotsalainen 2010	(96)	Blood	Finland	6	8(40%)	28.0±6.2	38.6±6.6
Sajadi 2013	(97)	Peripheral blood	Iran	6	41(41.0%)	NR	40±9
Samaras 2010	(98)	Paired of subcutaneous (SAT) and visceral adipose tissue(VAT)	Australia	8	NR	35.0±3.2	62±8
Sathishkumar 2016	(99)	Blood	India	8	16(64%)	26.6±4.3	45.0±9
Saukkonen 2018	(100)	Venous blood	Finland	7	NR	27.9±3.5	62.1±0.7
Shah 2011	(101)	Blood	USA	5	NR	NR	NR
Sindhu 2016	(102)	Plasma	Kuwait	7	NR	32.68±4.63	50.92±6.42
Sindhu 2017	(12)	Plasma	Kuwait	7	NR	32.68±4.63	50.92±6.42
Sozer 2014	(103)	Blood	Turkey	7	29(48.3%)	27.57±4.09	52.96±12.64
Tavangar 2016	(104)	Fasting blood	Iran	6	NR	NR	NR
Tavangar 2017	(105)	Salivary	Iran	6	NR	NR	NR
Toan 2018	(106)	Serum	Vietnam	7	29(58.0%)	26.7±5.3	59±4
Tokarz 2016	(107)	Blood	Poland	6	29(60.4%)	31.18±5.21	63.02±9.92
Tvarijonaviciute 2017	(108)	Salivary	Spain	5	14(45.2%)	26.4±5.4	49.8±20.9
Umapathy 2018	(109)	Fasting blood	India	8	NR	27.14±3.35	54.07±11.09
Wada 2000	(110)	Serum	Japan	5	32(71.1%)	NR	61.1±4.2
Wang 2019	(111)	Whole blood	China	5	48(69.57%)	25.61±5.76	64.29±3.77
Wei 2013	(112)	Venous blood	China	6	14(70.0%)	25.19±4.01	60.6±9.61
Wender 2008	(11)	Venous blood	Poland	6	0(0.0%)	21.5±9.0	28.8±1.5
Wu 2014	(113)	Blood	China	7	82(45.5%)	25.85±2.60	52.58±5.96
Xu 2015	(114)	Forearm venous blood	China	8	36(72.0%)	24.77±2.67	56.83±12.48
Yadav 2017	(115)	Blood	UK	6	NR	53±9	NR
Yang 2012	(116)	Venous blood	China	8	15(53.5%)	22.4±3.1	52±8
Yi 2014	(117)	Blood	China	8	20(52.6%)	23.65±2.89	59.52±14.14
Zeng 2019	(118)	Vitreous humor	China	7	10(50.0%)	NR	55.63±7.64
Zhang 2015	(119)	Abdominal omental adipose tissues	China	6	3(50.0%)	36.27±2.97	50±6
Zhou 2016	(120)	Blood	China	7	14(36.7%)	25.4±5.4	59.0±10.5

NR, not report; BMI, Body Mass Index; USA, United States of America; UK, United Kingdom.

Considering sample types of chemokines, 62 eligible studies measured chemokines from peripheral blood samples, 13 from serum samples, 10 from plasma samples, and 13 from other samples. Additionally, the included studies were conducted in China (23), India (9), Italy (6), Japan (6), Germany (5), Iran (5), Turkey (4), USA (4), Brazil (3), Poland (3), South Korea (3), Spain (3), and other regions (24). Also, 69 studies used ELISA to determine chemokines, 10 studies used Luminex, and 19 studies used other methods. The study quality assessment indicated that NOS scores varied between 5 and 8, with 55 studies classified as moderate quality and 44 classified as high quality.

### Main Outcomes

**Table 2** shows the subgroup analysis of chemokines concentrations between T2DM patients and controls; and between PDM participants and controls. Thus, as regards CC chemokines, CCL1 concentrations were compared between 20 T2DM patients and 50 controls. Therefore, concentrations of CCL1 were significantly higher in patients with T2DM than in controls (SMD=0.69; 95% CI: 0.28 - 1.09; **Figure 1**). Besides, no significant heterogeneity was observed in the included studies (*I²=*0). Further, CCL2 concentrations were compared between 3,856 T2DM patients and 2,958 controls, and the results indicated that CCL2 concentrations were in patients with T2DM than in the controls (SMD=1.51; 95% CI: 1.15 - 1.88; **Figure 1**), but with significant heterogeneity (*I²=*97%). Also, the CCL4 concentrations were compared between 829 T2DM patients and 411 controls. The results showed higher concentrations of CCL4 in T2DM patients than in the controls (SMD=1.14; 95% CI: 0.51 - 1.78; **Figure 1**), but with significant heterogeneity (*I²=*94%). Similarly, the concentrations of CCL5 were compared between 800 T2DM patients and 884 controls, and it was found that these concentrations were higher in T2DM patients than in the controls (SMD=1.42; 95% CI: 0.56 - 2.29; **Figure 1**), but with significant heterogeneity (*I²=*98%). In addition, CCL11 concentrations were compared between 621 T2DM patients and 793 controls, and the results showed that T2DM patients had higher concentrations of CCL11 than the controls (SMD=0.77; 95% CI: 0.20 - 1.34; **Figure 1**), but with significant heterogeneity (*I²=*95%).

**Table 2:** Subgroup analysis of chemokines between T2DM and PDM participants and controls.

	N	SMD	95%-CI		Heterogeneity		
					Q	τ²	I²
**CC chemokines in T2DM**							
CCL1	2	0.69	0.28	1.09	0.02	0.00	0.00%
CCL11	10	0.77	0.20	1.34	191.59	0.78	95.30%
CCL13	1	0.38	-0.24	1.01	0.00	--	--
CCL15	1	1.26	0.58	1.95	0.00	--	--
CCL19	2	-0.08	-1.33	1.18	5.11	0.66	80.40%
CCL2	59	1.51	1.15	1.88	2123.05	1.93	97.30%
CCL20	2	2.09	-0.55	4.72	42.62	3.53	97.70%
CCL21	1	0.58	-0.06	1.21	0.00	--	--
CCL22	2	-0.54	-2.89	1.82	38.18	2.82	97.40%
CCL23	3	0.33	-0.12	0.79	2.56	0.04	22.00%
CCL24	2	0.34	-2.01	2.70	29.10	2.78	96.60%
CCL25	1	0.40	-0.23	1.03	0.00	--	--
CCL26	1	0.76	0.12	1.40	0.00	--	--
CCL27	1	0.82	0.17	1.47	0.00	--	--
CCL3	10	1.18	-0.07	2.44	534.93	3.78	98.30%
CCL4	11	1.14	0.51	1.78	178.98	1.03	94.40%
CCL5	16	1.42	0.56	2.29	670.89	2.90	97.80%
CCL7	2	-1.83	-5.43	1.76	71.34	6.62	98.60%
CCL8	1	0.84	0.19	1.49	0.00	--	--
**CXC chemokines in T2DM**							
CX3CL1	7	1.45	0.42	2.48	237.68	1.86	97.5%
CXCL1	5	1.48	-0.86	3.83	406.74	7.08	99.0%
CXCL10	19	0.87	0.32	1.42	643.86	1.41	97.2%
CXCL11	2	2.81	-2.19	7.80	67.38	12.78	98.5%
CXCL12	8	0.60	-0.89	2.08	683.75	4.46	99.0%
CXCL13	1	0.61	-0.03	1.25	0.00	--	--
CXCL16	2	1.97	-2.27	6.21	58.43	9.20	98.3%
CXCL2	1	0.00	-0.62	0.62	0.00	--	--
CXCL4	1	0.78	0.37	1.18	0.00	--	--
CXCL5	1	-0.74	-1.39	-0.10	0.00	--	--
CXCL6	1	1.09	0.42	1.75	0.00	--	--
CXCL8	32	1.18	0.64	1.72	1172.62	2.25	97.4%
CXCL9	3	0.05	-0.86	0.97	12.25	0.54	83.7%
**Chemokines in PDM**							
CCL11	1	0.00	-0.19	0.18	0.00	--	--
CCL2	5	-0.11	-0.19	-0.03	4.17	0.00	4.10%
CCL5	2	0.79	-0.04	1.61	25.81	0.34	96.10%
CX3CL1	1	1.50	0.96	2.03	0.00	--	--
CXCL10	3	0.10	-0.57	0.77	59.20	0.34	96.60%
CXCL12	1	0.78	0.57	0.98	0.00	--	--
CXCL8	5	0.09	-0.91	1.09	210.62	1.22	98.10%

Heterogeneity was quantified using I^2^ and its significance was tested using the Q statistics. SMD, standardized mean difference; DF, degrees of Freedom; T2DM, Type-2 diabetes mellitus; PDM, prediabetes.

Considering CXC chemokines, concentrations of CXCL8 were compared between 1,405 T2DM patients and 1,599 controls, and the results indicated that T2DM patients had higher concentrations of CXCL8 than the controls (SMD=1.18; 95% CI: 0.64 - 1.72; **Figure 1**), but with significant heterogeneity (*I²=*97%). Additionally, CXCL10 concentrations were compared between 1,116 T2DM patients and 1,461 controls, and the results showed that the concentrations of CXCL10 were higher in patients with T2DM than the controls (SMD=0.87; 95% CI: 0.32 - 1.42; **Figure 1**), but with significant heterogeneity (*I²=*97%). Also, the concentrations of CX3CL1 were compared between 539 T2DM patients and 510 controls, and the results showed higher concentrations of CX3CL1 in patients with T2DM than in the controls (SMD=1.45; 95% CI: 0.42 - 2.48; **Figure 1**) but with significant heterogeneity (*I²=*97%). However, when all chemokines concentrations, taken together, were compared between 3,005 PDM patients and 3,541 controls, no significant difference was observed (SMD=0.20; 95% CI: -0.09 - 0.50; **Figure 2**). Nevertheless, when specific chemokines concentrations were considered, there were lower concentrations of CCL2 in patients with PDM than in the controls (SMD=-0.11; 95% CI: -0.19 - -0.03; **Figure 2**); but there was no significant difference in the concentrations of the following chemokines between patients with PDM and the controls: CCL5 (SMD=0.79; 95% CI: -0.04 - 1.61; **Figure 2**), CXCL8 (SMD=0.09; 95% CI: -0.91 - 1.09; **Figure 2**), and CXCL10 (SMD=0.10; 95% CI: -0.57 - 0.77; **Figure 2**). In addition, funnel plots indicated low probability of publication bias (**Appendix 7**). Moreover, sensitivity analysis indicated that any single study influenced little change in the SMD of chemokines concentrations in PDM and T2DM. **Appendix 8** shows in more detail results of the analysis of the chemokines concentration differences between patients with T2DM or PDM and the controls.

Considering subgroups according to gender, the results indicated that concentrations of chemokines in the female T2DM patients (SMD=1.11; 95% CI: 0.79 - 1.43; **Table 3**) and male T2DM patients (SMD=1.11; 95% CI: 0.87 - 1.35; **Table 3**) were significantly higher than in the controls. However, no significant difference was observed in the male PDM participants (SMD=-0.02; 95% CI: -0.41 - 0.37; **Table 3**). Furthermore, subgroup analysis indicated that the concentrations of chemokines were higher in the over 60 years old with T2DM than in their counterparts forming the control group (SMD=1.39; 95% CI: 1.12 - 1.65; **Table 3**). Similarly, the concentrations of chemokines were higher in the under 60 years old with T2DM than in their counterparts forming the control group (SMD=0.95; 95% CI: 0.69 - 1.22; **Table 3**) in T2DM, but with a lower effect value (SMD) than in the over 60 years old group. However, no significant difference was observed in the PDM participants as regards to age differences. Considering methods for measuring chemokines (Luminex method and ELISA method subgroups), subgroup analysis indicated that the T2DM patients had higher concentrations of chemokines than the controls, when chemokines were measured using the Luminex method (SMD=0.71; 95% CI: 0.14 - 1.28; **Table 3**) and the ELISA method (SMD=1.32; 95% CI:1.05 - 1.59; **Table 3**). However, no significant difference was observed between the PDM participants and the controls when the ELISA method was used (SMD=0.17; 95% CI: -0.20 - 0.55; **Table 3**).

**Table 3:** Subgroup analysis of chemokines between T2DM and PDM participants and controls.

Subgroup		SMD	95%-CI		Heterogeneity		
					Q	τ²	I²
**T2DM**							
**Gender**	Female	1.11	0.79	1.43	4524.88	2.76	0.98
	Male	1.11	0.87	1.35	3172.76	1.37	0.97
**Age**	>60	1.39	1.12	1.65	1572.77	1.11	0.96
	≤60	0.95	0.69	1.22	6193.05	2.43	0.98
**Method**	ELlSA	1.32	1.05	1.59	5246.49	2.05	0.98
	Luminex	0.71	0.14	1.28	1854.35	3.04	0.98
	Other	0.95	0.71	1.19	586.75	0.74	0.90
**PDM**							
**Gender**	Female	0.55	0.22	0.88	36.38	0.15	0.84
	Male	-0.02	-0.41	0.37	494.19	0.43	0.98
**Age**	>60	0.24	0.02	0.51	146.96	0.17	0.94
	≤60	0.19	0.41	0.79	339.84	0.70	0.98
**Method**	ELISA	0.17	-0.20	0.55	538.33	0.45	0.98
	Luminex	0.23	0.06	0.39	6.06	0.01	0.34

Subgroup analyses are performed to compare the concentration of chemokines between the T2DM and PDM and the controls. Heterogeneity was quantified using I^2^ and its significance was tested using the Q statistics. NR, not report; T2DM, Type-2 diabetes mellitus; PDM, prediabetes; ELISA, Enzyme linked immunosorbent assay; SMD, standardized mean difference.

## Discussion

To the best of our knowledge, this is the first systematic review and meta-analysis to explore the association between chemokines concentrations and T2DM/PDM. We identified and systematically reviewed 98 studies. We found that the concentrations of CC chemokines (e.g. CCL1, CCL2, CCL4, CCL5 and CCL11), CXC chemokines (e.g. CXCL8 and CXCL10) and CX3CL1 chemokines were significantly higher in patients with T2DM than in the controls. However, no significant difference in these concentrations was observed between patients with PDM and the controls. These results suggest that some chemokines may play a key role in modulating the pathological process of the progression of PDM to T2DM. Exploring the role of unique chemokines, produced from the immune system, could help to provide a novel therapeutic target for T2DM.

Inflammation appears to be involved in the interplay among chemokines and progression of T2DM, as this has been shown in other several pathological conditions (121–124). Moreover, recent reviews suggested that chemokines, CXCL10, CXCL9, and CXCL11, are implicated in the pathogenesis of autoimmune diseases such as autoimmune thyroiditis, type 1 diabetes, Graves disease, Thyroid eye disease, and Addison’s disease (125–128). Also, evidence indicates CCL2 and CXCL10 chemokine modulations by cytokines and PPARγ agonist in Graves’ ophthalmopathy (129). In this regard, PPARγ agonist activation plays an inhibitory role on CXCL10, but stimulates the release of CCL2. Moreover, a recent systematic review and meta-analysis suggested that chemokines, CCL3, CCL4, CCL5, CCL20, CXCL8 and CXCL11, are implicated in the pathogenesis of non-alcoholic fatty liver disease, post-traumatic stress disorder, and also different types of cancers (130–133).

The chemokines system plays a variety of roles in the T2DM microenvironment. Pancreatic islets and peri-pancreatic adipose tissue (PAT) are exposed to an early damage by genetic or environmental factors and start to secrete numerous pro-inflammatory chemokines. The chemokines and their receptors can also cause a variety of immune cells to enter the pancreatic islets and PAT site to play the role of immune attack. Pro-inflammatory chemokines are bound to their receptors; hence activating the nuclear factor-kappaB (NF-κB/IκBα), and Adenosine 5-monophosphate-activated protein kinase (AMPK) activation pathway, which stimulates a proinflammatory condition. Free fatty acid may also activate inflammatory pathways that may lead to DNA damage.

Moreover, dysfunction of free fatty acid can lead to superoxide and subsequent hydrogen peroxide, which can combine with nitric oxide, such as peroxynitrite (ONOO-), to create peroxynitrite. The compromised mitochondrial electron transport chain (ETC) and the reducing adenosine triphosphate (ATP) synthesis will further lead to the production of reactive oxygen species/reactive nitrogen species (ROS/RNS).

**Figure 3:**
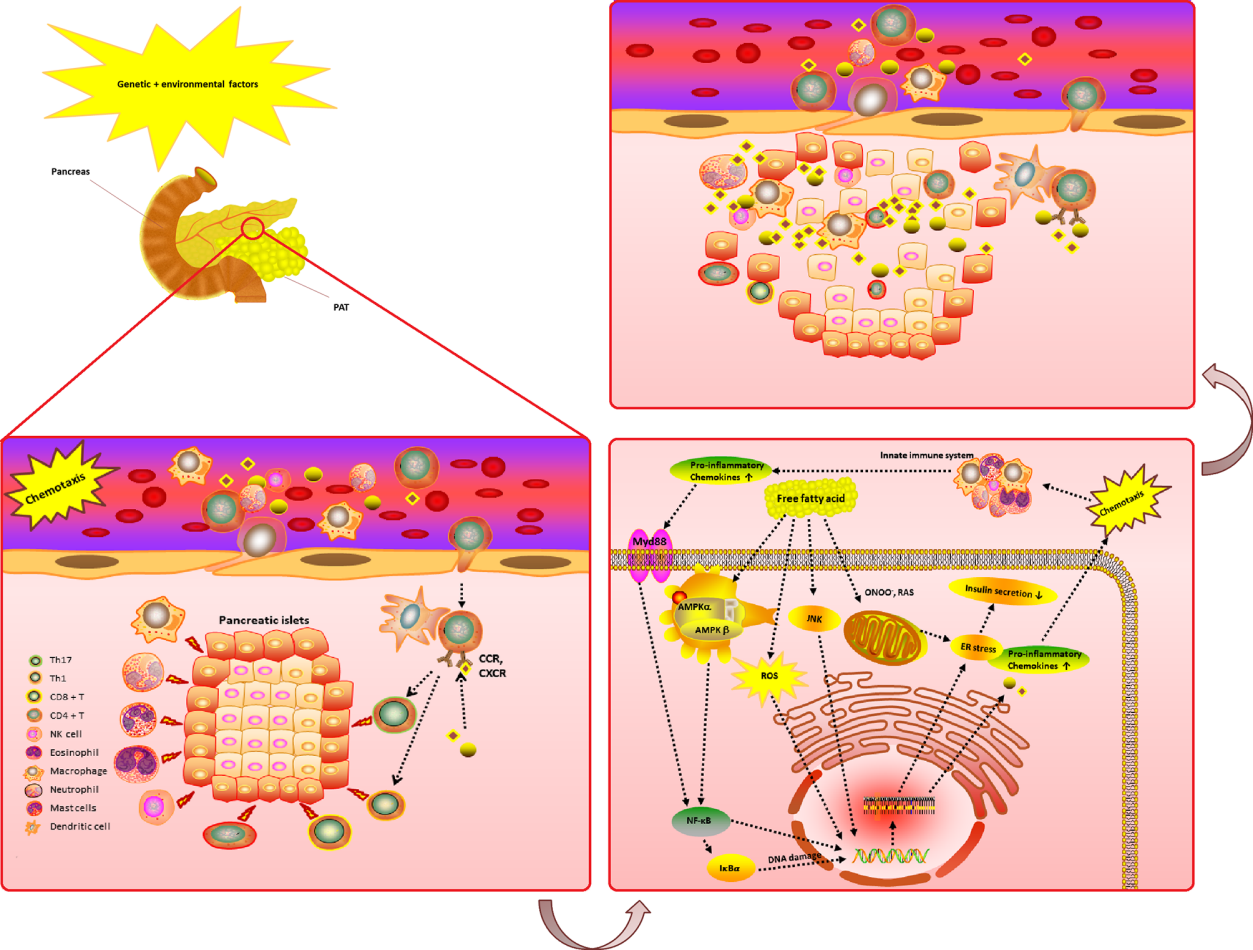
The complicated chemokines and their receptors network in the microenvironment of T2DM. The chemokine system plays a variety of roles in the T2DM microenvironment. Pancreatic islets and PAT are exposed to an early damage by genetic or environmental factors and start to secrete numerous pro-inflammatory chemokines. The chemokines and their receptors can also cause a variety of immune cells to enter the pancreatic islets and PAT site to play the role of immune attack. Pro-inflammatory chemokines are bind to their receptors activating the NF-κB/IκBα and AMPK activation pathway, which stimulates a proinflammatory condition. Free fatty acid may also activate inflammatory pathways and DNA damage. As a result of the Free fatty acid dysfunction, superoxide and subsequently hydrogen peroxide generation (which can combine with nitric oxide, an example of ONOO-, to create peroxynitrite, such as ROS/RNS) may occur due to compromised mitochondrial ETC and reducing ATP synthesis. All these processes impact ER stress, leading to a reduction in the ability to secrete insulin. Moreover, T2DM progression is characterized by progressive secretion of pro-inflammatory chemokines/cytokines caused by β cell damage. Due to this process, various immune cell types (i.e., neutrophils, macrophages, NK cell, dendritic cell and specifically T cells) are recruited in the pancreatic tissue. These immune cells further release more innate inflammatory cytokines, which contribute to a rapid increase β cell death. T2DM, Type 2 diabetes mellitus; ROS, reactive oxygen species; RNS, reactive nitrogen species; NF-κB/IκBα, nuclear factor-kappaB; ATP, adenosine triphosphate; PAT, peri-pancreatic adipose tissue; CCR, CC chemokines receptor; CXCR, CXC chemokines receptor; ETC, electron transport chain; AMPK, Adenosine 5-monophosphate activated protein-kinase; ER, endoplasmic reticulum; Protein kinase B (AKT); ONOO-, peroxynitrite; GLUT, glucose transporters; NK, Natural killer; Tregs, Regulatory T cells. (Drawn by AK.).

All these processes impact the endoplasmic reticulum (ER) stress, leading to a reduction in the ability to secrete insulin. Moreover, T2DM progression is characterized by progressive secretion of pro-inflammatory chemokines/cytokines caused by β cell damage. Due to this process, various immune cell types (i.e., neutrophils, macrophages, Natural killer (NK) cell, dendritic cell and specifically T cells) are recruited in the pancreatic tissue. These immune cells further release more innate inflammatory cytokines, which contribute to β cell death (**Figure 3**).

Further, studies have shown that the progression of T2DM is often accompanied by chronic silent inflammation (6, 134). Thus, various pro-inflammatory chemokines mediators are involved in the pathogenesis of T2DM, and this may further alter the normal structure of β-cells by inducing pancreatic islet’s apoptosis (134). Therefore, it may be suggested that the association between T2DM and chemokines may be bidirectional (7).

Referring to higher concentrations of CCL1 in patients with T2DM than in controls, a previous study suggested that CCL1 attracts monocytes, macrophages, Th2 cells and Treg cells by interacting with cell surface chemokine receptor CCR8 (135). Among these monocytes, macrophages release a variety of proinflammatory cytokines (IL-1β and IL-6), which can be released into the systemic circulation (136). Besides, IL-1β can induce inflammation by binding to interleukin-1 receptor type I, which reduces the expression of insulin receptor substrate-1 (IRS-1) at the ERK-dependent transcriptional level and ERK-independent post-transcriptional level, resulting in impaired insulin secretion of islet β cells (137). Moreover, the mechanism of IR induced by IL-6 is complex in that it could not only suppress the lipoprotein lipase that consecutively increase the plasma levels of triglycerides, but could also prevent the metabolism of non-oxidative glucose (138). This is consistent with our result that CCL1 may play a decisive role during immunoregulation in the pathogenesis of T2DM.

Furthermore, in relation to the findings of this study, CCL2, CCL4, CCL5 and CCL11 are considered as pro-inflammatory chemokines. For example, the CCL2 is a chemokine specifically for CCR2 receptors, whereas the CCL4, CCL5 and CCL11 exert a wide range of activities through the CCR5 receptors (139). Among the various chemokine receptors, CCR2, and CCR5 are the most important receptors that play a central role in the pathogenesis of T2DM, and this is consistent with our results. Also, Szalai et al. found that a polymorphism in CCR2 expression has been associated with the progression of IR in children (140). In addition, it has been found that fat cells can secrete CCR2 in an inactive form. Activated CCR2 can induce the expression of various inflammatory genes and reduce insulin-dependent glucose uptake. Further, adipocytes can also secrete CCL2 and CCL3, which is an effective signal for macrophage recruitment (141).

Also, upregulation of CCL2 and CCL3 by adipocytes may contribute to the progression of IR in adipose tissue and peripheral tissues. Although the results showed lower concentrations of CCL2 in PDM than in the controls, the effect value was low. Therefore, future studies need to further explore the dose-response relationship and specific pathophysiological role of CCL2 in the pathogenesis, severity and progression of PDM. In addition, Chang et al. found that CCL4 and CCR5 might provide potential therapeutic targets in T2DM (142). With the dysfunction of β cells in T2DM, even before β cells are widely damaged, the CCL4 concentrations may rise ahead of time. Also, while there are β cells death and early islet graft loss, inflammatory stimuli with a CD40-CD40L interaction could induce the secretion of CCL4 through the Raf/MEK/ERK and NF-κB pathways in pancreatic islets (143). Therefore, CCL4 concentrations may be caused by the initial inflammatory damage of islet β cells.

As a member of the CXC chemokine family, the function of CXCL8 is to induce chemotaxis in its target cells, like T-cells, neutrophil granulocytes, basophils and adipocytes. There are many receptors capable of binding to CXCL8, but the most affinity to CXCL8 are receptors CXCR1 and CXCR2 (144). Clinical studies have shown that CXCL8 secreted by adipocytes may be related to complications, such as T2DM, which are related to the excess accumulation of intra-abdominal fat (145). Increasing evidence suggests that the intra-abdominal fat accumulation is closely related to decreased insulin sensitivity and increased T2DM pathophysiology (145, 146). These findings concur with the result of this study that T2DM patients have higher concentrations of CXCL8. Thus, CXCL8 may mediate the down regulation of adiponectin in obesity. Adiponectin can prevent the impairment of insulin signaling; hence CXCL8 may play a crucial and causal role in obesity-linked IR and T2DM (6).

Furthermore, as a member of the CXC chemokine family, CXCL10 is secreted by various cell types, such as monocytes, endothelial cells and fibroblasts. In this study, T2DM patients have been shown to have higher concentrations of CXCL10. This is possible because CXCL10 functions *via* chemokine receptor CXCR3 and toll-likereceptor 4 (TLR4) (97, 147). In addition, CXCL10 has been attributed to several roles, such as activating T-lymphocytes and monocytes/macrophages. Also, clinical studies have shown that the concentrations of CXCL10 secreted by patients with T2DM are higher than in the control group (97). Therefore, there is a hypothesis that the interferon gamma-inducible CXCL10 plays an important role in triggering β cell destruction. Also, CXCL10 can impair insulin secretion and decrease β cell viability. The specific mechanism may be that the CXCL10 induced β cells can sustain the activation of protein kinase B (Akt), c-Jun N-terminal kinase (JNK), and cleavage of p21-activated protein kinase 2 (PAK-2); hence switching Akt signals from proliferation to apoptosis. These effects were mediated by the TLR4 (147, 148).

As the only known member of the CX3C chemokine family, CX3CL1 has the chemoattractive activity for T cells, NK cells, and monocytes (149). Clinical studies have shown that plasma CX3CL1 is significantly reduced in the control group compared to patients with T2DM, which is consistent with our results (34). The CX3CL1 is known to mediate leukocyte chemotaxis, adhesion and survival, which causes chronic adipose inflammation, and is closely associated with T2DM. Specifically, CX3CL1 may play a key role in recruiting monocytes to adipose tissue, and then leads to systemic inflammation and IR (150). The influence of CX3CL1-mediated mechanism of leukocyte chemotaxis and adhesion might be a way that leads to T2DM and adipocyte dysfunction. Additionally, studies have shown that high CX3CL1 levels can lead to depletion of functional reserves of β cells and chronic hyperinsulinemia, while inhibition of CX3CL1/CX3CR1 system can regulate pancreatic islet β-cell function (149). For example, Rosiglitazone inhibits CX3CL1 expression and CX3CL1/CX3CR1 signaling by activating peroxisome proliferator-activated receptor γ (PPAR-γ), which can achieve the effect of treating IR and T2DM (151).

The subgroup results according to gender indicated that concentrations of chemokines were significantly higher in the T2DM female and male patients than in their respective control groups. However, no significant difference in these concentrations was observed between male PDM participants and controls. This result may suggest that gender differences are related to PDM. This is consistent with the results of some previous studies which suggested that gender hormones may play an important role in the pathogenesis of impaired fasting glucose (IFG) and impaired glucose tolerance (IGT) (152, 153). In general, IFG is more prevalent in males, whereas IGT is more prevalent in females (152, 154). Also, some studies on preclinical gender-related genetic differences have shown that chemokine gene polymorphisms are associated with gender (155, 156). Therefore, we conjecture that the high expression of chemokines in females in the early stage of diabetes may have an indirect effect on insulin resistance through pro-inflammatory effect, and may also play a direct role in impaired glucose tolerance affect by regulating metabolism, inflammation and insulin signaling pathways. Thus, gender-specific differences in the pathogenesis of PDM suggest consideration for personalized treatment of PDM according to gender.

Furthermore, subgroup analysis showed that, although the concentrations of chemokines were higher in both age groups with T2DM, the over 60 years old had a higher effect value (SMD) than the under 60 years. In fact, previous studies have shown that chemokines are deeply influenced by aging and age-related inflammation kinetics (157, 158). Moreover, these results suggest that the elderly cannot control excessive inflammatory state causing the pro-inflammatory cytokines (158–160). Therefore, we speculate that the high concentrations of chemokines with excessive inflammatory response in the elderly T2DM patients could be the response to the perturbations in their imbalanced immune microenvironment. How to improve this imbalanced immune microenvironment may be a novel issue that would indicate that therapies should target the immune microenvironment in the treatment of T2DM in the elderly (161).

In addition, subgroup analysis indicated that the T2DM patients had higher concentrations of chemokines than the controls, when chemokines were measured using the Luminex method and the ELISA method. However, no significant difference was observed between the PDM participants and the controls when the ELISA method was used. These results may suggest that there was a potential heterogeneity when chemokines detection method was ELISA, and Luminex method has higher sensitivity and specificity. Alternatively, in consisted with our results, some previous studies showed that the Luminex method is permit the rapid, simultaneous detection of multiple inflammatory factors and chemokines with high sensitivity and specificity (162, 163). Therefore, we recommend the use of Luminex method for chemokines detection in future studies.

This systematic review and meta-analysis has some strengths and limitations. For example, this study analyzed various chemokines using broad inclusion criteria. Therefore, the use of a comprehensive search strategy, with multiple variant names of chemokines when searching in the databases, is the main strength of this study. Besides, this study has helped to clarify the previously reported results on the association between chemokines and T2DM or PDM. Therefore, the findings might open up new perspectives in T2DM for early diagnosis, identification of novel biomarkers, and novel targets for pharmacological interventions.

However, the number of available studies on chemokines (e.g., CCL13, CCL15, CCL21, CXCL2 and CXCL6) is limited, which may lead to biased or imprecise results. Accordingly, future studies should pay more attention to the investigation of the role of chemokines in T2DM and PDM. Also, there was lack of the following information in the included original studies: BMI, blood pressure, smoking and drinking. Therefore, it was difficult to quantify various confounding factors at individual level. In this regard, confounding factors should be better reported in future studies. Further, the original data for this study came from cross-sectional or case-control studies, which may not make causal inference. Therefore, population-based cohort studies are needed in the future to establish causal relationships between chemokines and T2DM or PDM.

## Conclusions

This systematic review and meta-analysis suggests that the progression of PDM to T2DM may be associated with elevated concentrations of chemokines (CCL1, CCL2, CCL4, CCL5, CCL11, CXCL8, CXCL10 and CX3CL1). Understanding the complex interplay of chemokines and the progression of PDM to T2DM may facilitate the identification of potential targets in prevention, diagnosis, and treatment of T2DM. The value of these chemokines in clinical practice should be elucidated in further studies.

## Data Availability Statement

The original contributions presented in the study are included in the article/**Supplementary Material**, further inquiries can be directed to the corresponding author.

## Author Contributions 

XP and AL contributed to the study design, while SW and AK contributed to the data collection. Statistical analyses and interpretation of results were performed by XP and AK, whereas XP and SW drafted the manuscript and edited the language. All authors contributed to the article and approved the submitted version.

## Funding

The research is financially supported by Hunan Provincial Key Laboratory of Clinical Epidemiology and the Hunan Provincial Key Research and Development Program (2018SK2065), China.

## Conflict of Interest

The authors declare that the research was conducted in the absence of any commercial or financial relationships that could be construed as a potential conflict of interest.
