# Supplementary material for: Chemokines in Prediabetes and Type 2 Diabetes: A Meta-Analysis
Source: Front Immunol. 2021 May 13;12:622438. doi: 10.3389/fimmu.2021.622438 (PMC8161229; doi:10.3389/fimmu.2021.622438)
Supplement: Supplementary file 1 [file DataSheet_1.pdf]

Supplementary appendix

Contents

Appendix 1a: Electronic search strategies
Appendix 1b: Study selection flow chart

Appendix 2(a＆b): Characteristics of included studies

Appendix 3a: The classification of chemokines and their receptors
Appendix 3b: The distribution—cell type of chemokine receptors
Appendix 4: Forest plot of CC chemokines between T2DM patients and controls
Appendix 5: Forest plot of CXC chemokine receptors between T2DM patients and controls
Appendix 6: Forest plot of chemokine between PDM patients and controls
Appendix 7: Egger funnel plots of T2DM patients compared to controls

Appendices References

Appendix 1a: Electronic search strategies

Search	Query
1	chemokine*
2	ccl1 or ccl2 or ccl3 or ccl4 or ccl5 or ccl6 or ccl7 or ccl8 or ccl9 or ccl10 or ccl11 or ccl12 or ccl13 or ccl14 or ccl15 or ccl16 or ccl17 or ccl18 or ccl19 or ccl20 or ccl21 or ccl22 or ccl23 or ccl24 or ccl25 or ccl26 or ccl27 or ccl28
3	cxcl1 or cxcl2 or cxcl3 or cxcl4 or cxcl5 or cxcl6 or cxcl7 or cxcl8 or cxcl9 or cxcl10 or cxcl11 or cxcl12 or cxcl13 or cxcl14 or cxcl15 or cxcl16 or cxcl17
4	xcl1 or xcl2
5	cx3cl1
6	ccl or cxcl or xcl or cx3cl
7	scya1 or scya2 or scya3 or scya4 or scya5 or scya6 or scya7 or scya8 or scya9 or scya10 or scya11 or scya12 or scya13 or scya14 or scya15 or scya16 or scya17 or scya18 or scya19 or scya20 or scya21 or scya22 or scya23 or scya24 or scya25 or scya26 or scya27 or scya28
8	scyb1 or scyb2 or scyb3 or scyb4 or scyb5 or scyb6 or scyb7 or scyb8 or scyb9 or scyb10 or scyb11 or scyb12 or scyb13 or scyb14 or scyb15 or scyb16 or scyb17
9	scyc1 or scyc2
10	sycd1
11	scya or scyb or scyc or scyd
12	chemokine receptor*

13	ccr1 or ccr2 or ccr2b or ccr3 or ccr4 or ccr5 or ccr6 or ccr7 or ccr8 or ccr9 or ccr10
14	cxcr1 or cxcr2 or cxcr3 or cxcr3b or cxcr4 or cxcr5 or cxcr6 or cxcr7
15	xcr1
16	cx3cr1
17	ccr or cxcr or xcr or cx3cr
18	chemotactic cytokine* or chemokine*
19	i-309 or i309 or tca-3 or tca3 or sise
20	IL-8 or GCP-2 or CXCR1 or NAP-2 or ENA-78 or GROα or GROβ or GROγ or PF4 or IP-10 or MIG or I-TAC or SDF-1 or BCA-1 or SR-PSOX or BRAK or MCP-1 or MCP-4 or CCR2 or MCP-3 or MCP-2 or MIP-1β or MIP-1α or CCR5 or RANTES or MPIF-1 or T2DM-1 or T2DM-2 or T2DM-4 or Eotaxin or Eotaxin-2 or Eotaxin-3 or TARC or CCR4 or MDC or MIP-3α or ELC or CCR7 or SLC or I-309 or TECK or CTACK or MEC or PARC or Lymphotactin or XCR1 or SCM-1β or Fractalkine or Chemerin
21	S1 OR S2 OR S3 OR S4 OR S5 OR S6 OR S7 OR S8 OR S9 OR S10 OR S11 OR S12 OR S13 OR S14 OR S15 OR S16 OR S17 OR S18 OR S19 OR S20
22	Diabetes Mellitus, Non Insulin Dependent OR Diabetes Mellitus, Non-Insulin-Dependent OR Non-Insulin-Dependent Diabetes Mellitus OR Diabetes Mellitus, Stable OR Stable Diabetes Mellitus OR Diabetes Mellitus, Type II OR NIDDM OR Diabetes Mellitus, Noninsulin Dependent OR Diabetes Mellitus, Maturity-Onset OR Diabetes Mellitus, Maturity Onset OR Maturity-Onset Diabetes Mellitus OR Maturity Onset Diabetes Mellitus OR T2DM OR

	Diabetes Mellitus, Slow Onset OR Slow-Onset Diabetes Mellitus OR Type 2 Diabetes Mellitus OR Noninsulin-Dependent Diabetes Mellitus OR Noninsulin Dependent Diabetes Mellitus OR Maturity-Onset Diabetes OR Diabetes, Maturity-Onset OR Maturity Onset Diabetes OR Type 2 Diabetes OR Diabetes, Type 2 OR Diabetes Mellitus, Adult-Onset OR Adult-Onset Diabetes Mellitus OR Diabetes Mellitus, Adult Onset
23	Diabetes Mellitus OR Diabetes Insipidus OR Diet, Diabetic OR Prediabetic State OR Scleredema Adultorum OR Glycation End Products, Advanced OR Glucose Intolerance OR Gastroparesis OR Diabetes Mellitus, Type 2 OR Diabetes Mellitus, Noninsulin-Dependent OR Diabetes Mellitus, Ketosis-Resistant OR Diabetes Mellitus, Ketosis Resistant OR Ketosis-Resistant Diabetes Mellitus
24	S22 OR S23
25	S21 AND S24

1386 of Web of Science

TITLE: (Diabetes Mellitus OR Diabetes Insipidus OR Diet, Diabetic OR Prediabetic State OR Scleredema Adultorum OR Glycation End Products, Advanced OR Glucose Intolerance OR Gastroparesis OR Diabetes Mellitus, Type 2 OR Diabetes Mellitus, Noninsulin-Dependent OR Diabetes Mellitus, Ketosis-Resistant OR Diabetes Mellitus, Ketosis Resistant OR Ketosis-Resistant Diabetes Mellitus OR Diabetes Mellitus, Non Insulin Dependent OR Diabetes Mellitus, Non-Insulin-Dependent OR Non-Insulin-Dependent Diabetes Mellitus OR Diabetes Mellitus, Stable OR Stable Diabetes Mellitus OR Diabetes Mellitus, Type II OR NIDDM OR Diabetes Mellitus, Noninsulin Dependent OR Diabetes Mellitus, Maturity-Onset OR Diabetes Mellitus, Maturity Onset OR Maturity-Onset Diabetes Mellitus OR Maturity Onset Diabetes Mellitus OR T2DM OR Diabetes Mellitus, Slow Onset OR Slow-Onset Diabetes Mellitus OR Type 2 Diabetes Mellitus OR Noninsulin-Dependent Diabetes Mellitus OR Noninsulin Dependent Diabetes Mellitus OR Maturity-Onset Diabetes OR Diabetes, Maturity-Onset OR Maturity Onset Diabetes OR Type 2 Diabetes OR Diabetes, Type 2 OR Diabetes Mellitus, Adult-Onset OR Adult-Onset Diabetes Mellitus OR Diabetes Mellitus, Adult Onset) AND TOPIC: (chemokine* or ccl1 or ccl2 or ccl3 or ccl4 or ccl5 or ccl6 or ccl7 or ccl8 or ccl9 or ccl10 or ccl11 or ccl12 or ccl13 or ccl14 or ccl15 or ccl16 or ccl17 or ccl18 or ccl19 or ccl20 or ccl21 or ccl22 or ccl23 or ccl24 or ccl25 or ccl26 or ccl27 or ccl28 or cxcl1 or cxcl2 or cxcl3 or cxcl4 or cxcl5 or cxcl6 or cxcl7 or cxcl8 or cxcl9 or cxcl10 or cxcl11 or cxcl12 or cxcl13 or cxcl14 or cxcl15 or cxcl16 or cxcl17 or xcl1 or xcl2 or cx3cl1 or ccl or cxcl or xcl or cx3cl or scya1 or scya2 or scya3 or scya4 or scya5 or scya6 or scya7 or scya8 or scya9 or scya10 or scya11 or scya12 or scya13 or scya14 or scya15 or

scya16 or scya17 or scya18 or scya19 or scya20 or scya21 or scya22 or scya23 or scya24 or scya25 or scya26 or scya27 or scya28 or scyb1 or scyb2 or scyb3 or scyb4 or scyb5 or scyb6 or scyb7 or scyb8 or scyb9 or scyb10 or scyb11 or scyb12 or scyb13 or scyb14 or scyb15 or scyb16 or scyb17 or scyc1 or scyc2 or sycd1 or scya or scyb or scyc or scyd or chemokine receptor* or ccr1 or ccr2 or ccr2b or ccr3 or ccr4 or ccr5 or ccr6 or ccr7 or ccr8 or ccr9 or ccr10 or cxcr1 or cxcr2 or cxcr3 or cxcr3b or cxcr4 or cxcr5 or cxcr6 or cxcr7 or xcr1 or cx3cr1 or ccr or cxcr or xcr or cx3cr or chemotactic cytokine* or chemokine* or i-309 or i309 or tca-3 or tca3 or sise or IL-8 or GCP-2 or CXCR1 or NAP-2 or ENA-78 or GROα or GROβ or GROγ or PF4 or IP-10 or MIG or I-TAC or SDF-1 or BCA-1 or SR-PSOX or BRAK or MCP-1 or MCP-4 or CCR2 or MCP-3 or MCP-2 or MIP-1β or MIP-1α or CCR5 or RANTES or MPIF-1 or T2DM-1 or T2DM-2 or T2DM-4 or Eotaxin or Eotaxin-2 or Eotaxin-3 or TARC or CCR4 or MDC or MIP-3α or ELC or CCR7 or SLC or I-309 or TECK or CTACK or MEC or PARC or Lymphotactin or XCR1 or SCM-1β or Fractalkine or Chemerin or Limphotactin or SCM-1 or C-10 or Mrp-1 or MIP-1γ or MRP2 or Eotaxin-1 or MCP-5 or Leukotactin-1 or MIP-5 or LEC or NCC-4 or MTN1 or MIP-4 or AMAC1 or ELC or MIP-3β or LARC or MIP-3 or 6Ckine or MPIF-2 or Eotaxin-2 or MIP-4α or CTAK or MGSA or MIP-2α or MIP-2β or PF-4 or BLC or Lungkine or SRPSOX)

3780 of Embase

('diabetes mellitus':ab,ti OR 'diabetes insipidus':ab,ti OR 'diet, diabetic':ab,ti OR 'prediabetic state':ab,ti OR 'scleredema adultorum':ab,ti OR 'glycation end products, advanced':ab,ti OR 'glucose intolerance':ab,ti OR gastroparesis:ab,ti OR 'diabetes mellitus, type 2':ab,ti OR

'diabetes mellitus, noninsulin-dependent':ab,ti OR 'diabetes mellitus, ketosis-resistant':ab,ti OR 'diabetes mellitus, ketosis resistant':ab,ti OR 'ketosis-resistant diabetes mellitus':ab,ti OR 'diabetes mellitus, non insulin dependent':ab,ti OR 'diabetes mellitus, non-insulin-dependent':ab,ti OR 'non-insulin-dependent diabetes mellitus':ab,ti OR 'diabetes mellitus, stable':ab,ti OR 'stable diabetes mellitus':ab,ti OR 'diabetes mellitus, type ii':ab,ti OR niddm:ab,ti OR 'diabetes mellitus, noninsulin dependent':ab,ti OR 'diabetes mellitus, maturity-onset':ab,ti OR 'diabetes mellitus, maturity onset':ab,ti OR 'maturity-onset diabetes mellitus':ab,ti OR 'maturity onset diabetes mellitus':ab,ti OR t2dm:ab,ti OR 'diabetes mellitus, slow onset':ab,ti OR 'slow-onset diabetes mellitus':ab,ti OR 'type 2 diabetes mellitus':ab,ti OR 'noninsulin-dependent diabetes mellitus':ab,ti OR 'noninsulin dependent diabetes mellitus':ab,ti OR 'maturity-onset diabetes':ab,ti OR 'diabetes, maturity-onset':ab,ti OR 'maturity onset diabetes':ab,ti OR 'type 2 diabetes':ab,ti OR 'diabetes, type 2':ab,ti OR 'diabetes mellitus, adult-onset':ab,ti OR 'adult-onset diabetes mellitus':ab,ti OR 'diabetes mellitus, adult onset':ab,ti) AND (ccl1:ab,ti OR ccl2:ab,ti OR ccl3:ab,ti OR ccl4:ab,ti OR ccl5:ab,ti OR ccl6:ab,ti OR ccl7:ab,ti OR ccl8:ab,ti OR ccl9:ab,ti OR ccl10:ab,ti OR ccl11:ab,ti OR ccl12:ab,ti OR ccl13:ab,ti OR ccl14:ab,ti OR ccl15:ab,ti OR ccl16:ab,ti OR ccl17:ab,ti OR ccl18:ab,ti OR ccl19:ab,ti OR ccl20:ab,ti OR ccl21:ab,ti OR ccl22:ab,ti OR ccl23:ab,ti OR ccl24:ab,ti OR ccl25:ab,ti OR ccl26:ab,ti OR ccl27:ab,ti OR ccl28:ab,ti OR cxcl1:ab,ti OR cxcl2:ab,ti OR cxcl3:ab,ti OR cxcl4:ab,ti OR cxcl5:ab,ti OR cxcl6:ab,ti OR cxcl7:ab,ti OR cxcl8:ab,ti OR cxcl9:ab,ti OR cxcl10:ab,ti OR cxcl11:ab,ti OR cxcl12:ab,ti OR cxcl13:ab,ti OR cxcl14:ab,ti OR cxcl15:ab,ti OR cxcl16:ab,ti OR cxcl17:ab,ti OR xcl1:ab,ti OR xcl2:ab,ti OR cx3cl1:ab,ti OR ccl:ab,ti OR cxcl:ab,ti OR xcl:ab,ti OR cx3cl:ab,ti OR

scya1:ab,ti OR scya2:ab,ti OR scya3:ab,ti OR scya4:ab,ti OR scya5:ab,ti OR scya6:ab,ti OR scya7:ab,ti OR scya8:ab,ti OR scya9:ab,ti OR scya10:ab,ti OR scya11:ab,ti OR scya12:ab,ti OR scya13:ab,ti OR scya14:ab,ti OR scya15:ab,ti OR scya16:ab,ti OR scya17:ab,ti OR scya18:ab,ti OR scya19:ab,ti OR scya20:ab,ti OR scya21:ab,ti OR scya22:ab,ti OR scya23:ab,ti OR scya24:ab,ti OR scya25:ab,ti OR scya26:ab,ti OR scya27:ab,ti OR scya28:ab,ti OR scyb1:ab,ti OR scyb2:ab,ti OR scyb3:ab,ti OR scyb4:ab,ti OR scyb5:ab,ti OR scyb6:ab,ti OR scyb7:ab,ti OR scyb8:ab,ti OR scyb9:ab,ti OR scyb10:ab,ti OR scyb11:ab,ti OR scyb12:ab,ti OR scyb13:ab,ti OR scyb14:ab,ti OR scyb15:ab,ti OR scyb16:ab,ti OR scyb17:ab,ti OR scyc1:ab,ti OR scyc2:ab,ti OR sycd1:ab,ti OR scya:ab,ti OR scyb:ab,ti OR scyc:ab,ti OR scyd:ab,ti OR 'chemokine receptor*':ab,ti OR ccr1:ab,ti OR ccr2b:ab,ti OR ccr3:ab,ti OR ccr6:ab,ti OR ccr8:ab,ti OR ccr9:ab,ti OR ccr10:ab,ti OR cxcr2:ab,ti OR cxcr3:ab,ti OR cxcr3b:ab,ti OR cxcr4:ab,ti OR cxcr5:ab,ti OR cxcr6:ab,ti OR cxcr7:ab,ti OR cx3cr1:ab,ti OR ccr:ab,ti OR cxcr:ab,ti OR xcr:ab,ti OR cx3cr:ab,ti OR 'chemotactic cytokine*':ab,ti OR chemokine*:ab,ti OR i309:ab,ti OR 'tca 3':ab,ti OR tca3:ab,ti OR sise:ab,ti OR 'il 8':ab,ti OR 'gcp 2':ab,ti OR cxcr1:ab,ti OR 'nap 2':ab,ti OR 'ena 78':ab,ti OR groα:ab,ti OR groβ:ab,ti OR groγ:ab,ti OR pf4:ab,ti OR 'ip 10':ab,ti OR mig:ab,ti OR 'i tac':ab,ti OR 'sdf 1':ab,ti OR 'bca 1':ab,ti OR 'sr psox':ab,ti OR brak:ab,ti OR 'mcp 1':ab,ti OR 'mcp 4':ab,ti OR ccr2:ab,ti OR 'mcp 3':ab,ti OR 'mcp 2':ab,ti OR 'mip 1β':ab,ti OR 'mip 1α':ab,ti OR ccr5:ab,ti OR rantes:ab,ti OR 'mpif 1':ab,ti OR 'T2DM 1':ab,ti OR 'T2DM 2':ab,ti OR 'T2DM 4':ab,ti OR eotaxin:ab,ti OR 'eotaxin 3':ab,ti OR tarc:ab,ti OR ccr4:ab,ti OR mdc:ab,ti OR 'mip 3α':ab,ti OR ccr7:ab,ti OR slc:ab,ti OR 'i 309':ab,ti OR teck:ab,ti OR ctack:ab,ti OR mec:ab,ti OR parc:ab,ti OR lymphotactin:ab,ti OR xcr1:ab,ti OR 'scm 1β':ab,ti

OR fractalkine:ab,ti OR chemerin:ab,ti OR limphotactin:ab,ti OR 'scm 1':ab,ti OR 'c 10':ab,ti OR 'mrp 1':ab,ti OR 'mip 1γ':ab,ti OR mrp2:ab,ti OR 'eotaxin 1':ab,ti OR 'mcp 5':ab,ti OR 'leukotactin 1':ab,ti OR 'mip 5':ab,ti OR lec:ab,ti OR 'ncc 4':ab,ti OR mtn1:ab,ti OR 'mip 4':ab,ti OR amac1:ab,ti OR elc:ab,ti OR 'mip 3β':ab,ti OR larc:ab,ti OR 'mip 3':ab,ti OR 6ckine:ab,ti OR 'mpif 2':ab,ti OR 'eotaxin 2':ab,ti OR 'mip 4α':ab,ti OR ctak:ab,ti OR mgsa:ab,ti OR 'mip 2α':ab,ti OR 'mip 2β':ab,ti OR 'pf 4':ab,ti OR blc:ab,ti OR lungkine:ab,ti OR srpsox:ab,ti)

2720 of Pubmed

((Diabetes Mellitus[Title/Abstract] OR Diabetes Insipidus[Title/Abstract] OR Diet, Diabetic[Title/Abstract] OR Prediabetic State[Title/Abstract] OR Scleredema Adultorum[Title/Abstract] OR Glycation End Products, Advanced[Title/Abstract] OR Glucose Intolerance[Title/Abstract] OR Gastroparesis[Title/Abstract] OR Diabetes Mellitus, Type 2[Title/Abstract] OR Diabetes Mellitus, Noninsulin-Dependent[Title/Abstract] OR Diabetes Mellitus, Ketosis-Resistant[Title/Abstract] OR Diabetes Mellitus, Ketosis Resistant[Title/Abstract] OR Ketosis-Resistant Diabetes Mellitus[Title/Abstract] OR Diabetes Mellitus, Non Insulin Dependent[Title/Abstract] OR Diabetes Mellitus, Non-Insulin-Dependent[Title/Abstract] OR Non-Insulin-Dependent Diabetes Mellitus[Title/Abstract] OR Diabetes Mellitus, Stable[Title/Abstract] OR Stable Diabetes Mellitus[Title/Abstract] OR Diabetes Mellitus, Type II[Title/Abstract] OR NIDDM[Title/Abstract] OR Diabetes Mellitus, Noninsulin Dependent[Title/Abstract] OR Diabetes Mellitus, Maturity-Onset[Title/Abstract] OR Diabetes Mellitus, Maturity

Onset[Title/Abstract] OR Maturity-Onset Diabetes Mellitus[Title/Abstract] OR Maturity Onset Diabetes Mellitus[Title/Abstract] OR T2DM[Title/Abstract] OR Diabetes Mellitus, Slow Onset[Title/Abstract] OR Slow-Onset Diabetes Mellitus[Title/Abstract] OR Type 2 Diabetes Mellitus[Title/Abstract] OR Noninsulin-Dependent Diabetes Mellitus[Title/Abstract] OR Noninsulin Dependent Diabetes Mellitus[Title/Abstract] OR Maturity-Onset Diabetes[Title/Abstract] OR Diabetes, Maturity-Onset[Title/Abstract] OR Maturity Onset Diabetes[Title/Abstract] OR Type 2 Diabetes[Title/Abstract] OR Diabetes, Type 2[Title/Abstract] OR Diabetes Mellitus, Adult-Onset[Title/Abstract] OR Adult-Onset Diabetes Mellitus[Title/Abstract] OR Diabetes Mellitus, Adult Onset[Title/Abstract])) AND (chemokine*[Title/Abstract] OR ccl1[Title/Abstract] OR ccl2[Title/Abstract] OR ccl3[Title/Abstract] OR ccl4[Title/Abstract] OR ccl5[Title/Abstract] OR ccl6[Title/Abstract] OR ccl7[Title/Abstract] OR ccl8[Title/Abstract] OR ccl9[Title/Abstract] OR ccl10[Title/Abstract] OR ccl11[Title/Abstract] OR ccl12[Title/Abstract] OR ccl13[Title/Abstract] OR ccl14[Title/Abstract] OR ccl15[Title/Abstract] OR ccl16[Title/Abstract] OR ccl17[Title/Abstract] OR ccl18[Title/Abstract] OR ccl19[Title/Abstract] OR ccl20[Title/Abstract] OR ccl21[Title/Abstract] OR ccl22[Title/Abstract] OR ccl23[Title/Abstract] OR ccl24[Title/Abstract] OR ccl25[Title/Abstract] OR ccl26[Title/Abstract] OR ccl27[Title/Abstract] OR ccl28[Title/Abstract] OR cxcl1[Title/Abstract] OR cxcl2[Title/Abstract] OR cxcl3[Title/Abstract] OR cxcl4[Title/Abstract] OR cxcl5[Title/Abstract] OR cxcl6[Title/Abstract] OR cxcl7[Title/Abstract] OR cxcl8[Title/Abstract] OR cxcl9[Title/Abstract] OR cxcl10[Title/Abstract] OR cxcl11[Title/Abstract] OR

cxcl12[Title/Abstract] OR cxcl13[Title/Abstract] OR cxcl14[Title/Abstract] OR cxcl15[Title/Abstract] OR cxcl16[Title/Abstract] OR cxcl17[Title/Abstract] OR xcl1[Title/Abstract] OR xcl2[Title/Abstract] OR cx3cl1[Title/Abstract] OR ccl[Title/Abstract] OR cxcl[Title/Abstract] OR xcl[Title/Abstract] OR cx3cl[Title/Abstract] OR scya1[Title/Abstract] OR scya2[Title/Abstract] OR scya3[Title/Abstract] OR scya4[Title/Abstract] OR scya5[Title/Abstract] OR scya6[Title/Abstract] OR scya7[Title/Abstract] OR scya8[Title/Abstract] OR scya9[Title/Abstract] OR scya10[Title/Abstract] OR scya11[Title/Abstract] OR scya12[Title/Abstract] OR scya13[Title/Abstract] OR scya14[Title/Abstract] OR scya15[Title/Abstract] OR scya16[Title/Abstract] OR scya17[Title/Abstract] OR scya18[Title/Abstract] OR scya19[Title/Abstract] OR scya20[Title/Abstract] OR scya21[Title/Abstract] OR scya22[Title/Abstract] OR scya23[Title/Abstract] OR scya24[Title/Abstract] OR scya25[Title/Abstract] OR scya26[Title/Abstract] OR scya27[Title/Abstract] OR scya28[Title/Abstract] OR scyb1[Title/Abstract] OR scyb2[Title/Abstract] OR scyb3[Title/Abstract] OR scyb4[Title/Abstract] OR scyb5[Title/Abstract] OR scyb6[Title/Abstract] OR scyb7[Title/Abstract] OR scyb8[Title/Abstract] OR scyb9[Title/Abstract] OR scyb10[Title/Abstract] OR scyb11[Title/Abstract] OR scyb12[Title/Abstract] OR scyb13[Title/Abstract] OR scyb14[Title/Abstract] OR scyb15[Title/Abstract] OR scyb16[Title/Abstract] OR scyb17[Title/Abstract] OR scyc1[Title/Abstract] OR scyc2[Title/Abstract] OR sycd1[Title/Abstract] OR scya[Title/Abstract] OR scyb[Title/Abstract] OR scyc[Title/Abstract] OR scyd[Title/Abstract] OR chemokine receptor*[Title/Abstract] OR ccr1[Title/Abstract] OR ccr2[Title/Abstract] OR

ccr2b[Title/Abstract] OR ccr3[Title/Abstract] OR ccr4[Title/Abstract] OR ccr5[Title/Abstract] OR ccr6[Title/Abstract] OR ccr7[Title/Abstract] OR ccr8[Title/Abstract] OR ccr9[Title/Abstract] OR ccr10[Title/Abstract] OR cxcr1[Title/Abstract] OR cxcr2[Title/Abstract] OR cxcr3[Title/Abstract] OR cxcr3b[Title/Abstract] OR cxcr4[Title/Abstract] OR cxcr5[Title/Abstract] OR cxcr6[Title/Abstract] OR cxcr7[Title/Abstract] OR xcr1[Title/Abstract] OR cx3cr1[Title/Abstract] OR ccr[Title/Abstract] OR cxcr[Title/Abstract] OR xcr[Title/Abstract] OR cx3cr[Title/Abstract] OR chemotactic cytokine*[Title/Abstract] OR chemokine*[Title/Abstract] OR i-309[Title/Abstract] OR i309[Title/Abstract] OR tca-3[Title/Abstract] OR tca3[Title/Abstract] OR sise[Title/Abstract] OR IL-8[Title/Abstract] OR GCP-2[Title/Abstract] OR CXCR1[Title/Abstract] OR NAP-2[Title/Abstract] OR ENA-78[Title/Abstract] OR GROα[Title/Abstract] OR GROβ[Title/Abstract] OR GROγ[Title/Abstract] OR PF4[Title/Abstract] OR IP-10[Title/Abstract] OR MIG[Title/Abstract] OR I-TAC[Title/Abstract] OR SDF-1[Title/Abstract] OR BCA-1[Title/Abstract] OR SR-PSOX[Title/Abstract] OR BRAK[Title/Abstract] OR MCP-1[Title/Abstract] OR MCP-4[Title/Abstract] OR CCR2[Title/Abstract] OR MCP-3[Title/Abstract] OR MCP-2[Title/Abstract] OR MIP-1β[Title/Abstract] OR MIP-1α[Title/Abstract] OR CCR5[Title/Abstract] OR RANTES[Title/Abstract] OR MPIF-1[Title/Abstract] OR T2DM-1[Title/Abstract] OR T2DM-2[Title/Abstract] OR T2DM-4[Title/Abstract] OR Eotaxin[Title/Abstract] OR Eotaxin-2[Title/Abstract] OR Eotaxin-3[Title/Abstract] OR TARC[Title/Abstract] OR CCR4[Title/Abstract] OR MDC[Title/Abstract] OR MIP-3α[Title/Abstract] OR ELC[Title/Abstract] OR CCR7[Title/Abstract] OR

SLC[Title/Abstract] OR I-309[Title/Abstract] OR TECK[Title/Abstract] OR CTACK[Title/Abstract] OR MEC[Title/Abstract] OR PARC[Title/Abstract] OR Lymphotactin[Title/Abstract] OR XCR1[Title/Abstract] OR SCM-1β[Title/Abstract] OR Fractalkine[Title/Abstract] OR Chemerin[Title/Abstract] OR Limphotactin[Title/Abstract] OR SCM-1[Title/Abstract] OR C-10[Title/Abstract] OR Mrp-1[Title/Abstract] OR MIP-1γ[Title/Abstract] OR MRP2[Title/Abstract] OR Eotaxin-1[Title/Abstract] OR MCP-5[Title/Abstract] OR Leukotactin-1[Title/Abstract] OR MIP-5[Title/Abstract] OR LEC[Title/Abstract] OR NCC-4[Title/Abstract] OR MTN1[Title/Abstract] OR MIP-4[Title/Abstract] OR AMAC1[Title/Abstract] OR ELC[Title/Abstract] OR MIP-3β[Title/Abstract] OR LARC[Title/Abstract] OR MIP-3[Title/Abstract] OR 6Ckine[Title/Abstract] OR MPIF-2[Title/Abstract] OR Eotaxin-2[Title/Abstract] OR MIP-4α[Title/Abstract] OR CTAK[Title/Abstract] OR MGSA[Title/Abstract] OR MIP-2α[Title/Abstract] OR MIP-2β[Title/Abstract] OR PF-4[Title/Abstract] OR BLC[Title/Abstract] OR Lungkine[Title/Abstract] OR SRPSOX[Title/Abstract])

889 of Cochrane Library

chemokine* or ccl1 or ccl2 or ccl3 or ccl4 or ccl5 or ccl6 or ccl7 or ccl8 or ccl9 or ccl10 or ccl11 or ccl12 or ccl13 or ccl14 or ccl15 or ccl16 or ccl17 or ccl18 or ccl19 or ccl20 or ccl21 or ccl22 or ccl23 or ccl24 or ccl25 or ccl26 or ccl27 or ccl28 or cxcl1 or cxcl2 or cxcl3 or cxcl4 or cxcl5 or cxcl6 or cxcl7 or cxcl8 or cxcl9 or cxcl10 or cxcl11 or cxcl12 or cxcl13 or cxcl14 or cxcl15 or cxcl16 or cxcl17 or xcl1 or xcl2 or cx3cl1 or ccl or cxcl or xcl or cx3cl or scya1 or scya2 or scya3 or scya4 or scya5 or scya6 or scya7 or scya8 or scya9 or scya10 or

scya11 or scya12 or scya13 or scya14 or scya15 or scya16 or scya17 or scya18 or scya19 or scya20 or scya21 or scya22 or scya23 or scya24 or scya25 or scya26 or scya27 or scya28 or scyb1 or scyb2 or scyb3 or scyb4 or scyb5 or scyb6 or scyb7 or scyb8 or scyb9 or scyb10 or scyb11 or scyb12 or scyb13 or scyb14 or scyb15 or scyb16 or scyb17 or scyc1 or scyc2 or sycd1 or scya or scyb or scyc or scyd or chemokine receptor* or ccr1 or ccr2 or ccr2b or ccr3 or ccr4 or ccr5 or ccr6 or ccr7 or ccr8 or ccr9 or ccr10 or cxcr1 or cxcr2 or cxcr3 or cxcr3b or cxcr4 or cxcr5 or cxcr6 or cxcr7 or xcr1 or cx3cr1 or ccr or cxcr or xcr or cx3cr or chemotactic cytokine* or chemokine* or i-309 or i309 or tca-3 or tca3 or sise or IL-8 or GCP-2 or CXCR1 or NAP-2 or ENA-78 or GROα or GROβ or GROγ or PF4 or IP-10 or MIG or I-TAC or SDF-1 or BCA-1 or SR-PSOX or BRAK or MCP-1 or MCP-4 or CCR2 or MCP-3 or MCP-2 or MIP-1β or MIP-1α or CCR5 or RANTES or MPIF-1 or T2DM-1 or T2DM-2 or T2DM-4 or Eotaxin or Eotaxin-2 or Eotaxin-3 or TARC or CCR4 or MDC or MIP-3α or ELC or CCR7 or SLC or I-309 or TECK or CTACK or MEC or PARC or Lymphotactin or XCR1 or SCM-1β or Fractalkine or Chemerin or Limphotactin or SCM-1 or C-10 or Mrp-1 or MIP-1γ or MRP2 or Eotaxin-1 or MCP-5 or Leukotactin-1 or MIP-5 or LEC or NCC-4 or MTN1 or MIP-4 or AMAC1 or ELC or MIP-3β or LARC or MIP-3 or 6Ckine or MPIF-2 or Eotaxin-2 or MIP-4α or CTAK or MGSA or MIP-2α or MIP-2β or PF-4 or BLC or Lungkine or SRPSOX in Title Abstract Keyword AND Diabetes Mellitus OR Diabetes Insipidus OR Diet, Diabetic OR Prediabetic State OR Scleredema Adultorum OR Glycation End Products, Advanced OR Glucose Intolerance OR Gastroparesis OR Diabetes Mellitus, Type 2 OR Diabetes Mellitus, Noninsulin-Dependent OR Diabetes Mellitus, Ketosis-Resistant OR Diabetes Mellitus, Ketosis Resistant OR Ketosis-Resistant Diabetes

Mellitus OR Diabetes Mellitus, Non Insulin Dependent OR Diabetes Mellitus, Non-Insulin-Dependent OR Non-Insulin-Dependent Diabetes Mellitus OR Diabetes Mellitus, Stable OR Stable Diabetes Mellitus OR Diabetes Mellitus, Type II OR NIDDM OR Diabetes Mellitus, Noninsulin Dependent OR Diabetes Mellitus, Maturity-Onset OR Diabetes Mellitus, Maturity Onset OR Maturity-Onset Diabetes Mellitus OR Maturity Onset Diabetes Mellitus OR T2DM OR Diabetes Mellitus, Slow Onset OR Slow-Onset Diabetes Mellitus OR Type 2 Diabetes Mellitus OR Noninsulin-Dependent Diabetes Mellitus OR Noninsulin Dependent Diabetes Mellitus OR Maturity-Onset Diabetes OR Diabetes, Maturity-Onset OR Maturity Onset Diabetes OR Type 2 Diabetes OR Diabetes, Type 2 OR Diabetes Mellitus, Adult-Onset OR Adult-Onset Diabetes Mellitus OR Diabetes Mellitus, Adult Onset in Title Abstract Keyword

Appendix 1b: Study selection flow chart. A flow chart demonstrating the selection process of articles included in the analysis as well as in the qualitative summary.

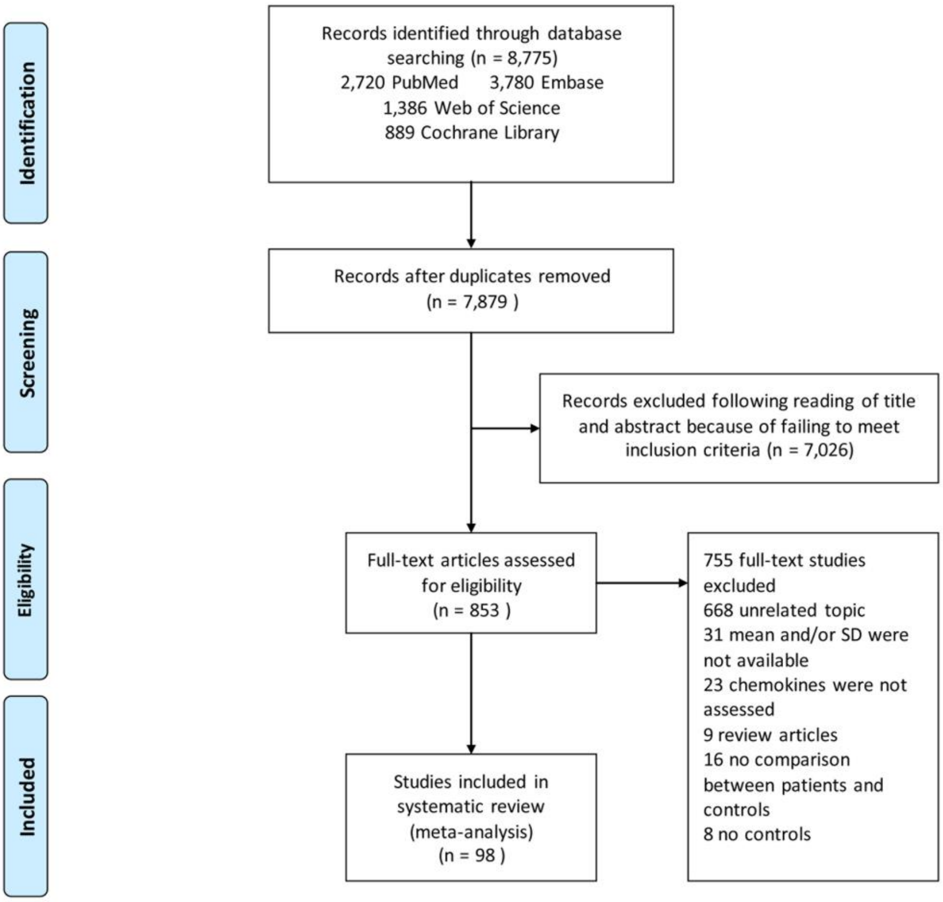

Appendix 2a: Characteristics of included studies

Study		Methods	Duration of diabetes (years)	Waist circumference (cm)	Waist-to-hip ratio	SBP (mmHg)	DBP (mmHg)	FPG	2h Postprandial blood glucose
Adela 2019	[1]	ELlSA	2	NR	NR	136.2 (127.5–149.6)	82.8 (78.2–93.0)	NR	NR
Afarideh 2016	[2]	ELlSA	NR	90.2	NR	127.5	80	8.44(mmol/L)	NR
Ahmed 2018	[3]	ELISA	7.09±0.29	NR	NR	NR	NR	197.79±3.22(mg/dL)	NR
Alicka 2019	[4]	ELISA	NR	NR	NR	NR	NR	NR	NR
AlmeidaPititto 2015	[5]	ELISA	NR	92.2(11.5)	NR	121(15)	78(10)	106.6(mg/dL)(8.1)	157.2(13.5)(mg/dL)
Alvarado 2018	[6]	ELISA	7.1(6.7)	NR	NR	NR	NR	NR	NR
Aravindhan 2018	[7]	ELISA	NR	NR	NR	122±20	82±10	195±73(mg/dL)	NR
Bala 2010	[8]	ELISA	7.5±0.8	NR	0.97±0.02 [0.8 – 1.17]	NR	NR	158±18(mg/dL)	NR
Baldane 2018A	[9]	ELISA	NR	NR	NR	128±13	81±11	140(88–388)(mg/dL)	NR
Baldane 2018B	[10]	ELISA	NR	NR	NR	122±8	73±6	6.05±0.89 (mmol/L)	14.6±5.52 (mmol/L)
Barchetta 2017	[11]	ELISA	7.6±7.6	96±6.9	NR	124±13.8	79±8	130±38.2(mmol/L)	NR
Cañizales 2018	[12]	ELISA	9.39±6.54	NR	0.94±0.06	NR	NR	4.87±0.79(mmol/L)	NR
Capone 2015	[13]	ELISA	NR	NR	NR	NR	NR	145.9±12.3(mg/dL)	NR

Cha 2012	[14]	WB	NR	NR	NR	135.5±21.9	81.3±16.5	7.13±3.45(mmol/L)	10.34±4.82(mmol/L)
Chang 2015	[15]	ELISA	NR	NR	NR	137.83±20.95	84.0 (79.0–90.0)	NR	NR
Chao 2010	[16]	ELISA	7.2±4.3	NR	NR	138.7±16.8	80.1±14.4	147±56(mg/dL)	NR
Chen 2017	[17]	ELISA	8.5±3.8	NR	NR	NR	NR	7.5±1.8	NR
Cheng 2012	[18]	ELISA	5.44±1.80	93.24±6.73	0.91±0.04	131.48±11.15	79.52±8.22	7.47±2.86(mmol/L)	11.47±1.87mmol/L
Cheung 2012	[19]	Multiplex	10	NR	NR	156.8±23.7	73.8±13.0	9.0±2.8(mmol/L)	NR
Cimini 2017	[20]	ELISA	7.1±6.3	113.7±12.8	NR	130.2±16.4	81.7±9.7	132.1±38.1(mg/dL)	NR
Cimini 2018	[21]	ELISA	NR	103.4±13.5	NR	132.5±15.1	81.8±9.4	142.7±50.6(mg/dL)	NR
Danielsson 2005	[22]	ELISA	NR	NR	NR	145(110–180)	NR	NR	NR
Davi 2009	[23]	ELISA	9.5±3.9	NR	NR	153±16	92±20	12.5±3.5(mmol/L)	NR
Defast 2000	[24]	ELISA	10±9	NR	NR	NR	NR	NR	NR
Degirmenci 2019	[25]	ELISA	NR	NR	NR	NR	NR	139.84±51.18(mg/dL)	NR
Derakhshan 2012	[26]	ELISA	9±3	NR	NR	NR	NR	140–190(mg/dL)	NR
Elmesallamy 2011	[27]	ELISA	7.53±1.61	NR	NR	NR	NR	NR	NR
Feng 2016	[28]	ELISA	NR	NR	NR	125.00±7.65	74.24±8.74	7.43±1.01(mmol/L)	NR
Funatsu 2009	[29]	ELISA	18.8±5.1	NR	NR	NR	NR	NR	NR
Geerlings 2000	[30]	ELISA	2.8±1.5	NR	NR	NR	NR	NR	NR
Giulietti 2006	[31]	Q-PCR	8.8	NR	NR	NR	NR	142(54–209) (mg/dL)	NR

Gokulakrishnan 2015	[32]	ELISA	1.5	74.1±7.1	NR	119±11	75±9	143±51(mg/dL)	NR
Gómez 2008	[33]	ELISA	8.7±6.1	NR	0.92±0.08	135.0±14.6	88.4±9.2	9.8±3.4(mmol/L)	NR
Gong 2016	[34]	ELISA	NR	NR	NR	NR	NR	7.83(6.53,10.32)(mmol/L)	NR
Hamid 2016	[35]	ELISA	12.54±4.11	NR	NR	121.50±1.12	80.67±1.01	9.35±0.28(mmol/L)	NR
Hara 2016	[36]	CL	NR	NR	NR	NR	NR	105.30±15.87 (mg/dL)	NR
He 2014	[37]	CL	1	NR	NR	NR	NR	8.6(5.9-12.1)(mmol/L)	NR
Herder 2005	[38]	ELISA	9.1±6.7	NR	0.94±0.08	146±21	84±11	NR	NR
Herder 2008	[39]	ELISA	9.1±6.7	NR	0.94±0.08	146±21	84±11	128(114-142)(mg/dL)	NR
Hernández 2008	[40]	ELISA	19	NR	NR	NR	NR	NR	NR
Hirsch 2012	[41]	ELISA	9.6(2.6)	NR	0.89(0.2)	NR	NR	NR	NR
Hu 2012	[42]	ELISA	6.54±4.71	NR	0.85±0.09	138.29±16.17	86.56±19.56	10.31±1.86(mmol/L)	NR
Huang 2012	[43]	RIA	1.4	NR	NR	NR	NR	NR	NR
Inayat 2019	[44]	Q-PCR	7	NR	NR	130[100–160]	80[59–109]	242[130–385](mmol/L)	NR
Kalninova 2014	[45]	ELISA	14.00±8.10	102(96–109)	NR	148(130–158)	81(74–85)	9.90±2.90(mmol/L)	NR
Kang 2010	[46]	ELISA	NR	NR	NR	138.2±21.6	82.1±14.1	7.32±3.23(mmol/L)	10.41±5.81(mmol/L)
Kou 2018	[47]	ELISA	NR	NR	NR	148(133-163)	83(74-95)	5.86(5.05-7.14)(mmol/L)	NR
Kumar 2012	[48]	ELISA	NR	NR	NR	NR	NR	220.3±47.9(mg/dL)	NR
Kumar 2013	[49]	ELISA	4.5	NR	NR	115 (82–152)	70 (59–90)	287	NR

								(200–653)(mg/dL)	
LandersRamos 2019	[50]	Luminex	NR	NR	NR	NR	NR	NR	NR
Lareyre 2018	[51]	ELISA	NR	NR	NR	NR	NR	NR	NR
Li 2019	[52]	ELISA	NR	90±8	NR	125±9	79±6	11.34±3.57 (mmol/L)	18.73±4.39 (mmol/L)
Liu 2011	[53]	Luminex	9.00±1.15	NR	NR	NR	NR	8.24±0.87 (mmol/L)	NR
Liu 2012	[54]	ELISA	5.16±7.17	NR	NR	136.43±22.49	80.27±9.81	8.83±3.36(mmol/L)	NR
Liuni 2015	[55]	Luminex	NR	NR	NR	NR	NR	142.6±23.28 (mg/dL)	NR
Lu 2017	[56]	ELISA	NR	85.27±9.08	NR	126.03±11.87	78.93±5.77	10.86±3.56 (mmol/L)	NR
Maegdefessel 2010	[57]	ELISA	NR	NR	NR	NR	NR	NR	NR
Maier 2008	[58]	ELISA	NR	NR	NR	NR	NR	NR	NR
Mangialardi 2019	[59]	ELISA	NR	NR	NR	NR	NR	NR	NR
McCarthy 2019	[60]	Luminex	NR	NR	NR	NR	NR	NR	NR
Mesia 2016	[61]	Luminex	NR	NR	NR	NR	NR	NR	NR
Mine 2008	[62]	IFT	NR	NR	NR	NR	NR	176±76 mg/dL	NR
Mohamed 2015	[63]	Luminex	9.67(1.70)	NR	NR	NR	NR	NR	NR
Murase 2012	[64]	ELISA	10.8±8.7	NR	NR	133±15	75±10	7.85± 1.98 (mmol/L)	NR
Nomura 2005	[65]	ELISA	NR	NR	NR	183±22	102±16	NR	NR
Omoto1 2015	[66]	ELISA	NR	NR	NR	NR	NR	102±21(mg/dL)	NR
Papatheodorou 2012	[67]	ELISA	10.9±8.5	107.7±11	1.02±0.07	144.9±23.8	85.8±10.6	179±53 mg/dL	NR

Pham 2012	[68]	Luminex	1.0(0.3)	NR	NR	133(117–145)	82(72–89)	NR	NR
Porta 2018	[69]	WB	17.00±4.00	NR	NR	NR	NR	149.6 [126.7,169.9](mg/dL)	NR
Prechel 2018	[70]	ELISA	NR	NR	NR	NR	NR	NR	NR
Pushpanathan 2016	[71]	ELISA	NR	NR	NR	NR	NR	NR	NR
Ruotsalainen 2010	[72]	ELISA	NR	NR	0.88±0.08	133±18	90±14	5.2±0.5(mmol/L)	8.7±0.8(mmol/L)
Sajadi 2013	[73]	ELISA	9±3	NR	NR	NR	NR	180±25 mg/dL	NR
Samaras 2010	[74]	Q-PCR	NR	114±15	NR	132±3	73±4	7.8±1.8 (mmol/L)	NR
Sathishkumar 2016	[75]	Q-PCR	NR	93±11	NR	135±21	81±11	189±46(mg/dL)	NR
Saukkonen 2018	[76]	Luminex	9.3(3.9)	NR	NR	142(16.7)	78.7 (8.1)	5.1(0.8)(mmol/L)	6.8(2.3) (mmol/L)
Shah 2011	[77]	Q-PCR	NR	NR	NR	NR	NR	NR	NR
Sindhu 2016	[78]	Luminex	NR	NR	NR	NR	NR	9.01±2.57(mmol/L)	NR
Sindhu 2017	[79]	Luminex	NR	NR	NR	NR	NR	9.01±2.57(mmol/L)	NR
Sozer 2014	[80]	ELISA	8.66±3.62	NR	NR	NR	NR	10.08±2.93(mmol/L)	NR
Tavangar 2016	[81]	ELISA	NR	NR	NR	NR	NR	26.6±152.8(mg/dL)	62.2±24.35(mg/dL)
Tavangar 2017	[82]	ELISA	NR	NR	NR	NR	NR	159.25±49.3(mg/dL)	210.2±44.8(mg/dL)
Toan 2018	[83]	ELISA	NR	NR	0.94[0.86–1.07]	NR	NR	7.65 [7–21.6](mmol/L)	NR
Tokarz 2016	[84]	ELISA	NR	NR	NR	NR	NR	10.15(7.40–12.00) (mmol/L)	NR

Tvarijonaviciute 2017	[85]	Multiplex	NR	NR	NR	NR	NR	NR	NR
Umapathy 2018	[86]	Multiplex	NR	NR	NR	128.8±14.2	83.59±12.4	176.6±54.29 (mg/dL)	211.7±47.4(mg/dL)
Wada 2000	[87]	ELISA	NR	NR	NR	NR	NR	NR	NR
Wang 2019	[88]	Q-PCR	NR	NR	NR	NR	NR	NR	NR
Wei 2013	[89]	ELISA	NR	NR	NR	NR	NR	8.57±4.42(mmol/L)	NR
Wender 2008	[90]	ELISA	11.0	NR	NR	NR	NR	6.04(4.4-8.8)(mmol/L)	NR
Wu 2014	[91]	ELISA	8.15±4.23	NR	NR	123.67±6.33	77.55±6.29	NR	NR
Xu 2015	[92]	ELISA	8.97±5.87	NR	NR	126.51±20.27	82.65±10.17	8.92±4.24(mmol/L)	15.45±5.14(mmol/L)
Yadav 2017	[93]	ELISA	NR	141(18)	NR	129(19)	71(90)	NR	NR
Yang 2012	[94]	ELISA	4.4±1.9	NR	NR	136±9	76±8	5.9±0.8(mmol/L)	NR
Yi 2014	[95]	ELISA	8.00±5.17	NR	0.9±0.05	150.61±18.87	83.68±9.41	7.91±2.57(mmol/L)	NR
Zeng 2019	[96]	Multiplex	10.87±3.18	NR	NR	141.21±15.19	77.84±10.83	NR	NR
Zhang 2015	[97]	Q-PCR	NR	NR	0.944±0.534	NR	NR	9.89±2.44(mmol/L)	NR
Zhou 2016	[98]	ELISA	NR	NR	NR	NR	NR	10.47±2.94(mmol/L)	NR

NR, not report; SBP, systolic blood pressure; DBP, diastolic blood pressure; FBP, fasting plasma glucose/fasting blood glucose.

Appendix 2b: Characteristics of included studies

Study	HbA1c	Creatinine	Uric acid	Cholesterol	TG	LDL	HDL	Adiponectin	CRP	Insulin	HOMA-I

Adela 2019	[1]	7.9(%)(7.2–9.1)	0.9 (0.7–1.0)(mg/dL)	4.0 (3.3–4.8)(mg/dL)	NR	NR	NR	NR	NR	NR	NR	NR
Afarideh 2016	[2]	7.9(%)	NR	NR	4.43(mmol/L)	NR	2.84(mmol/L)	1.09	NR	31.5(ng/ml)	NR	NR
Ahmed 2018	[3]	8.476(%)±0.15	NR	NR	257.69±4.76(mg/dL)	178.66±1.89(mg/dL)	181.14 ±1.99(mg/dL)	38.41±0.77 (mg/dL)	NR	NR	13.69±1.28(pmol/L)	6.69±0.62
Alicka 2019	[4]	NR	NR	NR	NR	NR	NR	NR	NR	NR	NR	NR
AlmeidaPititto 2015	[5]	NR	NR	NR	216.3 (38.6)(mg/dL)	160.7(92.1) (mg/dL)	132.9(mg/dL)(33.4)	52.0(mg/dL)(12.4)	10.1(mg/mL)	1.4(mg/L) (0.8–3.7)	5.7(mUI/mL)	NR
Alvarado 2018	[6]	8.8(%)(2)	NR	NR	NR	NR	NR	NR	NR	NR	NR	NR
Aravindhan 2018	[7]	9.2(%)±2.2	0.84±0.13(mg/dL)	NR	208±47(mg/dL)	NR	NR	NR	NR	NR	NR	NR
Bala 2010	[8]	7.5(%)±0.5	NR	NR	157 (mg/dL)±9	130(mg/dL)±11	88(mg/dL)±7	40(mg/dL) ±3	6.9±1.3(mg/mL)	22(mg/l)±4.8	NR	NR
Baldane 2018A	[9]	7.2(5.2–14.3)(%)	NR	NR	190±44(mg/dL)	160(mg/dL)	114±38(mg/dL)	45±11(mg/dL)	NR	NR	9.99(1.18–47.50)(mIU/mL)	NR
Baldane 2018B	[10]	NR	NR	NR	5.50±0.83(mmol/L)	2.22±1.13(mmol/L)	3.34±0.74 (mmol/L)	1.13±0.25 (mmol/L)	NR	3.79 (ng/mL)±1.28	NR	NR
Barchetta 2017	[11]	6.3(%)±0.5	0.97 (mg/dL)±0.3	5.8±0.9(mg/dL)	175.8±38(mg/dL)	134.6 (mg/dL)±70.8	NR	49.1(mg/dL)±13.3	6.7±3.5(mg/mL)	2.8±3.8(ng/mL)	10.7±5.5(mIU/mL)	4.2±1.8
Cañizales 2018	[12]	8.49±4.45(%)	0.047(mmol/L)±0.016	NR	5.06(mmol/L) ±1.00	2.35(mmol/L)±1.19	2.58(mmol/L)	1.39(mmol/L)±0.37	NR	NR	NR	NR

							±0.70					
Capone 2015	[13]	NR	NR	NR	NR	NR	NR	NR	NR	NR	NR	NR
Cha 2012	[14]	7.4±2.6(%)	73±33(μmol/L)	NR	5.12±0.94(mmol/L)	1.61±0.93(mmol/L)	NR	1.13±0.66(mmol/L)	NR	0.23±0.25 (mg/dL)	NR	4.5±3.5
Chang 2015	[15]	NR	NR	NR	209.50±31.58 mg/dL	159.5 (119.25–213.75) mg/dL	133.19 ±30.48 mg/dL	41.0(36.75–47.0)mg/dL	NR	NR	NR	NR
Chao 2010	[16]	6.9±1.8(%)	0.71±0.22 (mg/dL)	NR	181±46(mg/dL)	164±47(mg/dL)	49±12(mg/dL)	114±20(mg/dL)	NR	1.58±0.27 (mg/dL)	NR	NR
Chen 2017	[17]	8.8±3.5(%)	NR	NR	NR	NR	NR	NR	NR	NR	NR	NR
Cheng 2012	[18]	6.93±1.45%	72.56±16.07 μmol/L	NR	4.90±0.98mmol/L	1.56±0.65 mmol/L	2.93±0.81mmol/L	1.29±0.39mmol/L	NR	NR	NR	NR
Cheung 2012	[19]	NR	NR	NR	NR	NR	NR	NR	NR	NR	NR	NR
Cimini 2017	[20]	6.8±1.02%	NR	NR	178.6±37(mg/dL)	134.2±62.7 (mg/dL)	101.3±33.2(mg/dL)	50.3±14.7(mg/dL)	6.8±3.2(ng/mL)	3.05±3.92 mg/l	12.15±5.52(μU/L)	3.84±1.81
Cimini 2018	[21]	7.1±1.2%	NR	NR	185.1±26 mg/dL	114.2±61.4 (mg/dL)	115.4±23.1(mg/dL)	50.3±14.7(mg/dL)	5.4±3.3(ng/ml)	5.1±2.4(mg/L)	12±3.3(μU/L)	4.1±1.5
Danielsson 2005	[22]	6.75%(5.1–8.7)	91(79–174)(mol/L)	NR	5.25(3.3–7.35) (mmol/L)	1.22(0.4–4.6)(mmol/L)	2.9(1.7–4.6)(mmol/L)	1.2(0.5–2.5)(mmol/L)	NR	0.9(0.3–2.1)(mg/l)	NR	NR
Davi 2009	[23]	7.7±1.6%	NR	NR	5.9±1.0mmol/L	2.1±1.3mmol/L	NR	NR	NR	NR	NR	NR
Defast 2000	[24]	12±1(%)	NR	NR	NR	NR	NR	NR	NR	NR	NR	NR

Degirmenci 2019	[25]	7.24±1.99(%)	0.85±0.20 (mg/dL)	NR	NR	183.85±94.30(mg/dL)	139.20 ±22.81 (mg/dL)	NR	NR	NR	17.77±11.38(U/L)	NR
Derakhshan 2012	[26]	NR	NR	NR	170±5.7(mg/dL)	210±6(mg/dL)	140±6(mg/dL)	35±3(mg/dL)	NR	NR	NR	NR
Elmesallamy 2011	[27]	7.67±0.81%	0.62±0.043mg/dL	NR	168.59±7.23mg/dL	80.02±5.55 mg/dL	89.58±10.12 mg/dL	61.58±9.81 mg/dL	NR	6.54±1.45 mg/dL	NR	NR
Feng 2016	[28]	6.88±0.72%	76.10±17.29(μmol/L)	NR	4.62±0.66(mmol/L)	3.05±0.86(mmol/L)	2.80±0.53(mmol/L)	1.25±0.29(mmol/L)	NR	2.44±1.61 (mg/L)	NR	NR
Funatsu 2009	[29]	7.3±0.7 %	NR	NR	NR	NR	NR	NR	NR	NR	NR	NR
Geerlings 2000	[30]	8.7% (6-12.4)	NR	NR	NR	NR	NR	NR	NR	NR	NR	NR
Giulietti 2006	[31]	9.9(6.7–13.6)%	NR	NR	NR	NR	NR	NR	NR	NR	NR	NR
Gokulakrishnan 2015	[32]	7.9±2.2(%)	NR	NR	157±29(mg/dL)	126.2 (mg/dL)	93±25(mg/dL)	36±6(mg/dL)	3.8±0.17μg/mL	NR	26.7±11.2(IU/mL)	6.8±3.4
Gómez 2008	[33]	7.2±1.7 %	NR	NR	5.23±0.77(mmol/L)	1.45±1.05(mmol/L)	3.06±0.64(mmol/L)	1.53±0.35(mmol/L)	NR	3.35±3.27 (mg/l)	113.7±72.8(pmol/L)	7.20±6.04
Gong 2016	[34]	6.4(5.55,8.25)%	75(58.05-88.05)(mmol/L)	NR	4.56±1.11(mmol/L)	1.39(0.84,1.89)(mmol/L)	2.48±0.92 (mmol/L)	1.11±0.37(mmol/L)	NR	NR	NR	NR
Hamid 2016	[35]	9.51±0.29%	NR	NR	NR	NR	NR	NR	NR	NR	NR	NR

Hara 2016	[36]	5.72±0.44(%)	NR	NR	NR	NR	NR	NR	NR	NR	NR	NR
He 2014	[37]	NR	NR	NR	NR	NR	NR	NR	NR	NR	NR	4.2
Herder 2005	[38]	6.4(5.9-7.2)%	NR	6.1±1.5 (mg/dL)	235±4(mg/dL)	141(107;196)(mg/dL)	145±41(mg/dL)	50±14 (mg/dL)	NR	2.52(1.13; 5.63)(mg/l)	NR	4.54(2.65 61)
Herder 2008	[39]	6.4(5.9-7.2)%	NR	NR	235±4(mg/dL)	141(107;196)(mg/dL)	145±41(mg/dL)	50±14 (mg/dL)	NR	2.52(1.13; 5.63)(mg/l)	14.3(9.2-21.2)(mU/L)	4.54(2.65 61)
Hernández 2008	[40]	8.2±3.7%	NR	NR	NR	NR	NR	NR	NR	NR	NR	NR
Hirsch 2012	[41]	7.9(1.0)%	NR	3.9(0.2)(mg/dL)	185.9(5.6)(mg/dL)	105.6(9.2)(mg/dL)	NR	58.9(2.7)(mg/dL)	15.4(1.4)(ng/mL)	0.12(0.04)(mg/dL)	NR	1.2(0.1)
Hu 2012	[42]	7.22±1.28%	95.58±11.52(mmol/L)	NR	NR	NR	NR	NR	NR	NR	NR	NR
Huang 2012	[43]	NR	NR	NR	NR	NR	NR	NR	NR	NR	NR	NR
Inayat 2019	[44]	9.76[6–14]%	NR	NR	166[115–229](mg/dL)	176[126–223](mg/dL)	117[71–153](mg/dL)	35.20[24–48](mg/dL)	NR	NR	NR	NR
Kalninova 2014	[45]	5.02±0.71%	77.00±15.40(μmol/L)	NR	4.55±0.90(mmol/L)	1.57(1.2–2.2)(mmol/L)	2.49±0.70(mmol/L)	1.21±0.30(mmol/L)	NR	NR	NR	NR
Kang 2010	[46]	7.3±2.6%	72±22(μmol/L)	NR	5.12±0.98(mmol/L)	1.65±0.90(mmol/L)	NR	1.11±0.62 (mmol/L)	12.56±4.74(ng/l)	0.22±0.16 (mg/dL)	NR	4.3±2.5
Kou 2018	[47]	NR	65.90(53.48-81.73)(μmol/L)	NR	4.35(3.83-5.33)(mmol/L	1.71(1.28-2.16)(mmol/L)	2.73(2.18-3.53)(mmol/L)	1.17(1.00-1.40)(mmol/L)	8.42 pg/mL	NR	13.60(11.55-15.60)(mIU/L)	3.31(2.86 03)
Kumar 2012	[48]	8.3±0.7%	NR	NR	NR	NR	NR	NR	NR	NR	NR	NR

Kumar 2013	[49]	11.3(8.0 6)%	NR	NR	215 (124–259)mg/dL	182 (57–679)mg/dL	124(47–185) mg/dL	38(22–82) mg/dL	NR	NR	NR	NR
LandersRamos 2019	[50]	NR	NR	NR	NR	NR	NR	NR	NR	NR	NR	NR
Lareyre 2018	[51]	NR	NR	NR	NR	NR	NR	NR	NR	NR	NR	NR
Li 2019	[52]	9.65±2.41%	NR	NR	4.92±1.26 (mmol/L)	2.08 (1.38,3.08)(mmol/L)	2.51±1.06(mmol/L)	1.28±0.41(mmol/L)	NR	2.34(1.44, 5.09)(mg/l)	12.64 (10.58-15.36)(mU/L)	5.85 (4.41,8.5[illegible]
Liu 2011	[53]	9.89±0.68%	NR	NR	NR	NR	NR	NR	NR	NR	NR	NR
Liu 2012	[54]	9.5±2.8%	NR	NR	4.84±1.29(mmol/L)	2.51±1.75(mmol/L)	2.66±1.13(mmol/L)	1.24±0.40(mmol/L)	NR	NR	NR	NR
Liuni 2015	[55]	NR	NR	NR	NR	NR	NR	NR	NR	NR	NR	NR
Lu 2017	[56]	NR	NR	NR	NR	2.74±7.82(mmol/L)	NR	1.12±0.26(mmol/L)	NR	NR	NR	NR
Maegdefessel 2010	[57]	NR	NR	NR	151±50mg/dL	145±13mg/dL	79±38 mg/dL	39±22mg/dL	NR	10±16mg/L	NR	NR
Maier 2008	[58]	7.40±1.11%	NR	NR	NR	NR	NR	NR	NR	NR	NR	NR
Mangialardi 2019	[59]	10.4% 60±4	NR	NR	NR	NR	NR	NR	NR	NR	NR	NR
McCarthy 2019	[60]	NR	NR	NR	NR	NR	NR	NR	NR	NR	NR	NR
Mesia 2016	[61]	8.13±1.23%	NR	NR	NR	NR	NR	NR	NR	NR	NR	NR
Mine 2008	[62]	6.9±1.5%	NR	NR	211±50mg/dL	150±79mg/dL	NR	NR	NR	0.7±0.5mg/dL	NR	NR
Mohamed 2015	[63]	9.25 (0.49)%	NR	NR	NR	NR	NR	NR	NR	NR	NR	NR

Murase 2012	[64]	8.9±1.9%	NR	NR	NR	1.60±0.89 (mmol/L)	3.07±0.82 (mmol/L)	1.36±0.36(mmol/L)	NR	2.69±0.52 (mg/dL)	NR	NR
Nomura 2005	[65]	9.5±2.5%	NR	NR	6.24±1.34 (mmol/L)	2.39±0.67 (mmol/L)	NR	NR	NR	NR	NR	NR
Omoto1 2015	[66]	5.1±0.9%	0.62±0.19(mg/dL)	NR	220±28(mg/dL)	NR	133±32(mg/dL)	47±12(mg/dL)	4.54±1.01(μg/mL)	0.82±0.79 (mg/dL)	NR	NR
Papatheodorou 2012	[67]	8.07±1.52%	NR	5.2±1.5mg/dL	223±54mg/dL	200±217mg/dL	139±45mg/dL	49±13mg/dL	NR	NR	NR	NR
Pham 2012	[68]	NR	NR	NR	NR	NR	NR	NR	NR	NR	NR	NR
Porta 2018	[69]	NR	0.93[0.70, 1.16](mg/dL)	NR	193.00 (mg/dL)	99.00 [69.60, 140.20](mg/dL)	123.00 [89.00, 154.00](mg/dL)	74.00(mg/dL)	15.31[8.02, 20.46](μg/ml)	NR	6.67[2.54-9.52](uIU/ml)	2.48[0.90 3.21]
Prechel 2018	[70]	9.01%	NR	NR	171	184mg/dL	105mg/dL	38.5mg/dL	NR	NR	NR	NR
Pushpanathan 2016	[71]	8.56±2.68%	NR	NR	NR	NR	NR	NR	NR	NR	NR	NR
Ruotsalainen 2010	[72]	NR	NR	NR	NR	NR	NR	NR	NR	NR	Fasting plasma insulin: NGT 52.4±24.5 IGT 57.9±34.4 (pmol/L)、120 min plasma insulin NGT 238.92±53.4 IGT	NR

											261.72±58.5	
Sajadi 2013	[73]	NR	NR	NR	170±5.7(mg/dL)	210±6(mg/dL)	140±6(mg/dL)	35±3(mg/dL)	NR	NR	NR	NR
Samaras 2010	[74]	7.48±0.7%	NR	NR	4.6±0.4(mmol/L)	1.8±0.5(mmol/L)	2.6±0.5(mmol/L)	1.15±0.12(mmol/L)	7.7±4.2(μg/mL)	NR	20.5±3.0(pmol/L)	NR
Sathishkumar 2016	[75]	9.2±1.7%	NR	NR	170±48(mg/dL)	140±69(mg/dL)	103±37(mg/dL)	40±8(mg/dL)	NR	NR	NR	4.4±1.6
Saukkonen 2018	[76]	5.4(0.5)%	NR	NR	NR	1.3(0.6)(mmol/L)	NR	1.3(0.3)(mmol/L)	24.9±18.4(μg/mL)	NR	11.8(6.5)(pmol/L)	1.6(0.8)
Shah 2011	[77]	NR	NR	NR	NR	NR	NR	NR	NR	NR	NR	NR
Sindhu 2016	[78]	8.20±1.85%	NR	NR	5.03±1.00(mmol/L)	1.80±1.21(mmol/L)	3.12±1.13(mmol/L)	0.99±0.25(mmol/L)	NR	NR	NR	NR
Sindhu 2017	[79]	8.20±1.85%	NR	NR	5.03±1.00(mmol/L)	1.80±1.21(mmol/L)	3.12±1.13(mmol/L)	0.99±0.25(mmol/L)	NR	NR	NR	NR
Sozer 2014	[80]	7.25±1.66%	NR	NR	4.29±0.66(mmol/L)	1.73±0.33(mmol/L)	2.95±1.24(mmol/L)	1.27±0.23(mmol/L)	NR	NR	NR	NR
Tavangar 2016	[81]	NR	NR	NR	NR	NR	NR	NR	NR	NR	NR	NR
Tavangar 2017	[82]	NR	NR	NR	NR	NR	NR	NR	NR	NR	NR	NR
Toan 2018	[83]	7[5.4–14.4]%	69.5[38–123](μmol/L)	NR	5.0 [3.8–8.5](mmol/L)	1.8 [0.34-19.6](mmol/L)	3.0 [1.0–4.5](mmol/L)	1.2[0.7–4.6](mmol/L)	NR	NR	6.5[1.3-23.2](mIU/L)	2.3[0.4–9
Tokarz 2016	[84]	10.04(7.	90.54(80.54–	NR	4.69±1.29	1.67(1.13–	2.64±1	1.11(0.94–	NR	1.51(0.81	NR	NR

		35–8.45)%	108.56)(μmol/L)		(mmol/L)	2.42)(mmol/L)	.14 (mmol/L)	1.33)(mmol/L)		–2.98)(mg/l)		
Tvarijonaviciute 2017	[85]	NR	NR	NR	NR	NR	NR	NR	NR	NR	759.3pg/mL	NR
Umapathy 2018	[86]	11.54±2.27%	1.11±0.45(mg/dL)	NR	161.2±30.42(mg/dL)	171.0±38.49 (mg/dL)	136.0±28.21 (mg/dL)	39.51±10.6 (mg/dL)	NR	4.6(mg/l)	NR	NR
Wada 2000	[87]	NR	NR	NR	NR	NR	NR	NR	NR	NR	NR	NR
Wang 2019	[88]	8.22±2.64%	NR	NR	NR	NR	4.68±1.70(mmol/L)	NR	NR	NR	NR	NR
Wei 2013	[89]	8.05±2.10%	NR	NR	4.49±0.77(mmol/L)	2.07±1.70(mmol/L)	2.65±0.75(mmol/L)	0.94±0.28(mmol/L)	NR	8.87(mg/l)	11.48±4.65(mU/L)	4.77±3.2[illegible]
Wender 2008	[90]	7.04(4.8-10.0)%	NR	NR	NR	NR	NR	NR	NR	NR	NR	NR
Wu 2014	[91]	9.2(8.9–9.5)%	58.97±9.57(μmol/L)	NR	4.33(4.19–4.46)(mmol/L)	1.67(mmol/L)	3.26 (3.14–3.37)(mmol/L)	1.15±0.27(mmol/L)	NR	NR	NR	NR
Xu 2015	[92]	9.47±2.87%	NR	335.78±145.15(μmol/L)	5.75±1.78(mmol/L)	1.90±0.49 (mmol/L)	2.70–0.47(mmol/L)	1.01–0.30(mmol/L)	NR	NR	16.08–7.47(Ulu/mL)	8.87–7.8[illegible]
Yadav 2017	[93]	NR	NR	NR	4.36(0.80)(mmol/L)	1.98(1.00)(mmol/L)	2.26(0.61)(mmol/L)	1.18(0.22)(mmol/L)	1.54(0.66)(mg/l)	11.46(12.05)(mg/l)	NR	10.60(8.6[illegible]
Yang 2012	[94]	5.8±0.6%	68.1±14.6(μmol/L)	NR	4.2±0.8(mmol/L)	1.9±0.7(mmol/L)	2.2±0.6(mmo[illegible]	1.1±0.2(mmol/L)	NR	3.3±1.6(mg/l)	NR	NR

							l/L)					
Yi 2014	[95]	8.77±1.99%	NR	NR	5.12±1.22 (mmol/L)	2.16±1.21 (mmol/L)	NR	NR	NR	NR	NR	NR
Zeng 2019	[96]	7.18±1.02%	143±76.04(μmol/L)	NR	NR	NR	NR	NR	NR	NR	NR	NR
Zhang 2015	[97]	NR	NR	NR	NR	NR	NR	NR	NR	NR	NR	NR
Zhou 2016	[98]	NR	113±30 80±16(mmol/L)	333±88(μmol/L)	4.62±1.06(mmol/L)	2.31±1.34(mmol/L)	2.51±0.80(mmol/L)	1.22±0.34(mmol/L)	NR	5.76±2.01 (mg/L)	NR	NR

NR, not report; CRP, C-reactive protein; LDL, low-density lipoprotein; HDL, high-density lipoprotein; TG, triglycerides; and HOMA-IR, insulin resistance index.

Appendix 3a: The classification of chemokines and their receptors.

Subfamily of chemokine	Name of chemokine	Other names of chemokine	Receptors
**C chemokines**	XCL1	Limphotactin α, SCM-1α	XCR1
	XCL2	Limphotactin β, SCM-1β	XCR1
**CC chemokines**	CCL1	I-309	CCR8
	CCL2	MCP-1	CCR2
	CCL3	MIP-1α	CCR1, CCR5
	CCL4	MIP-1β	CCR5, CCR8
	CCL5	RANTES	CCR1, CCR3, CCR5
	CCL6	C-10, Mrp-1	CCR1
	CCL7	MCP-3	CCR1, CCR2, CCR3
	CCL8	MCP-2	CCR1, CCR2, CCR3, CCR5, CCR8
	CCL9	MIP-1γ, MRP2	CCR1,CCR3
	CCL11	Eotaxin-1	CCR3, CCR5
	CCL12	MCP-5	CCR2
	CCL13	MCP-4	CCR1, CCR2, CCR3, CCR5
	CCL14	T2DM-1	CCR1, CCR5
	CCL15	T2DM-2, Leukotactin-1, MIP-5	CCR1, CCR3
	CCL16	T2DM-4, LEC, NCC-4, MTN1	CCR1, CCR2, CCR5, CCR8

	CCL17	TARC	CCR4
	CCL18	MIP-4, AMAC1	CCR8
	CCL19	ELC, MIP-3β	CCR7
	CCL20	LARC, MIP-3α	CCR6
	CCL21	SLC,6Ckine	CCR7
	CCL22	MDC	CCR4
	CCL23	MPIF-1, MIP-3	CCR1, CCR3
	CCL24	MPIF-2, Eotaxin-2	CCR3
	CCL25	TECK	CCR9
	CCL26	Eotaxin-3, MIP-4α	CCR3, CX3CR1
	CCL27	CTAK	CCR10
	CCL28	MEC	CCR3, CCR10
**CXC chemokines**	CXCL1	GROα, MGSA	CXCR2
	CXCL2	GROβ, MIP-2α	CXCR2
	CXCL3	GROγ, MIP-2β	CXCR2
	CXCL4	PF-4	CXCR3
	CXCL5	ENA-78	CXCR2
	CXCL6	GCP-2	CXCR1, CXCR2
	CXCL7	NAP-2	CXCR2
	CXCL8	IL-8	CXCR1, CXCR2
	CXCL9	Mig	CXCR3
	CXCL10	IP-10	CXCR3
	CXCL11	I-TAC	CXCR3, CXCR7

	CXCL12	SDF-1	CXCR4, CXCR7
	CXCL13	BCA-1, BLC	CXCR5, CXCR3
	CXCL14	BRAK	Unknown
	CXCL15	Lungkine	Unknown
	CXCL16	SRPSOX	CXCR6
**CX3C chemokines**	CX3CL1	Fractalkine	CX3CR1

Appendix 3b: The distribution—cell type of chemokines receptors.

Receptors of Chemokine	Distribution—Cell Type
**CXCR1**	Neutrophils, monocytes, mast cells, basophils, dendric cells, CD8 T cells,natural killer cells
**CXCR2**	Neutrophils, monocytes, mast cells, basophils, dendric cells, natural killer cells
**CXCR3**	Basophils, Th1 cells, CD8 T cells, natural killer cells, Treg cells
**CXCR4**	Widely expressed
**CXCR5**	Basophils, CD8 T cells
**CXCR6**	Th1 cells, Th17 cells, natural killer cells, plasma cells
**CCR2**	Monocytes, macrophages, Th1 cells, basophil, natural killer cells
**CCR5**	Dendric cells, monocytes, macrophages, natural killer cells, Th1 cells, TH17 cells,
**CCR1**	Neutrophils, monocytes, macrophages, Th1 cells, basophils, dendric cells
**CCR3**	Eosinophils, basophils, Th2 cells, mast cells, dendric cells
**CCR4**	Th2 cells, Th17 cells, Treg cells, monocytes, basophils, CD4 & CD8 T cells
**CCR6**	Th17 cells, natural killer cells, Treg cells
**CCR7**	Dendric cells (mature), T cells, basophils
**CCR8**	Dendirc cells, monocytes, macrophages, Th2 cells, Treg cells
**CCR9**	Basophils, dendric cells
**CCR10**	T cells, IgA+ plasma cells
**XCR1**	Dendric cells
**CX3CR1**	Monocytes, macrophages, Th1 cells, dendric cells, natural killer cells

Appendix 4: Forest plot of CC chemokines between T2DM patients and controls

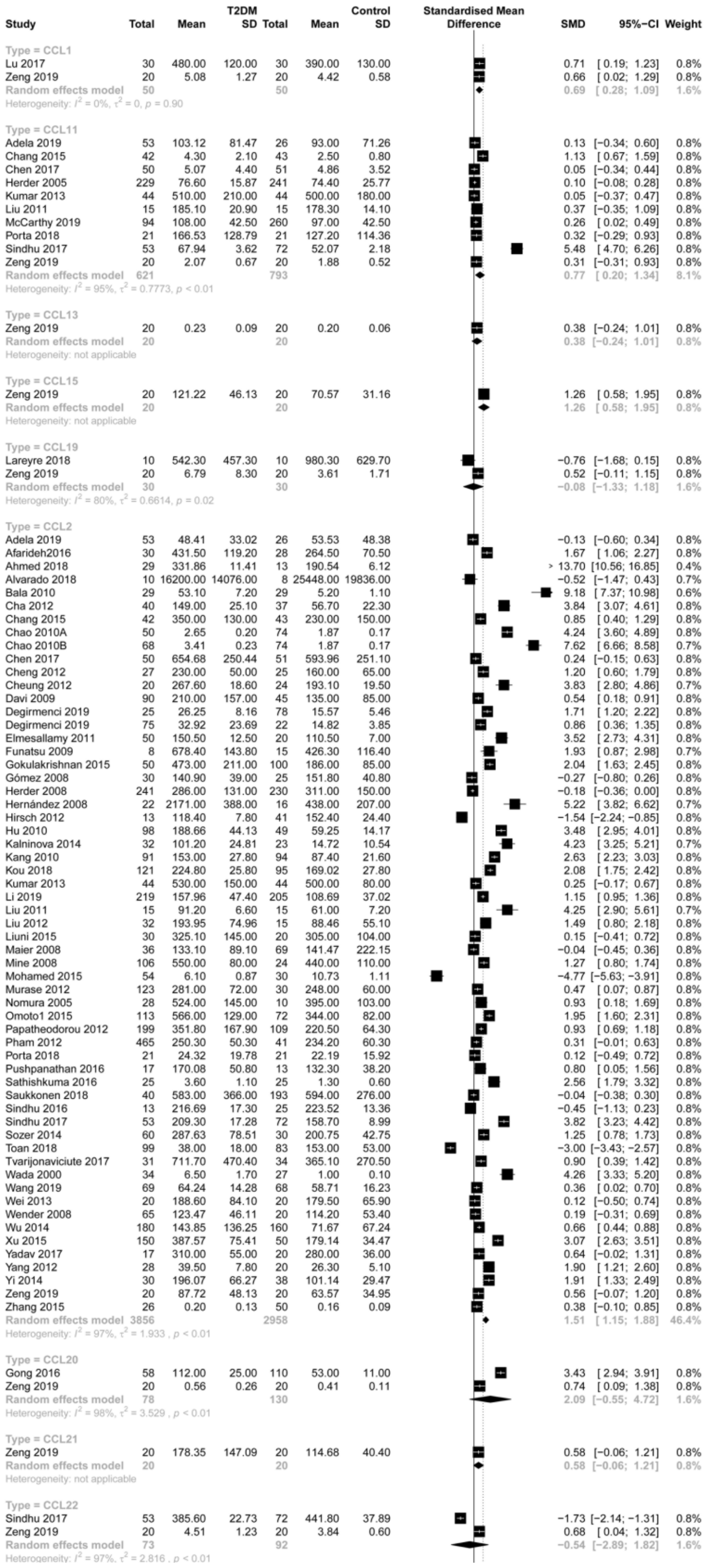

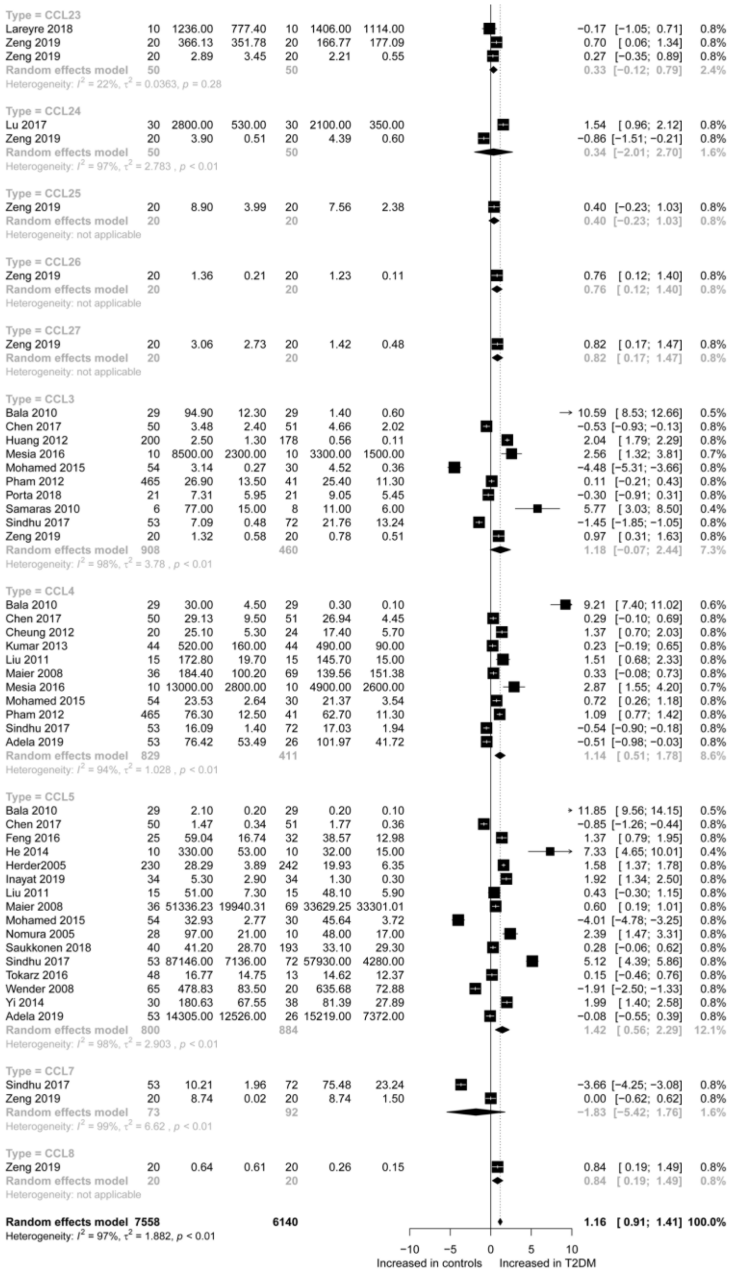

Study effect sizes of chemokines differences between T2DM and controls. Each data marker represents a study, and the size of the data marker is proportional to the total number of individuals in that study. The summary effect size for each chemokines is denoted by a diamond. T2DM, Type-2 diabetes mellitus; SMD, standardized mean difference.

Appendix 5: Forest plot of CXC chemokine between T2DM patients and controls

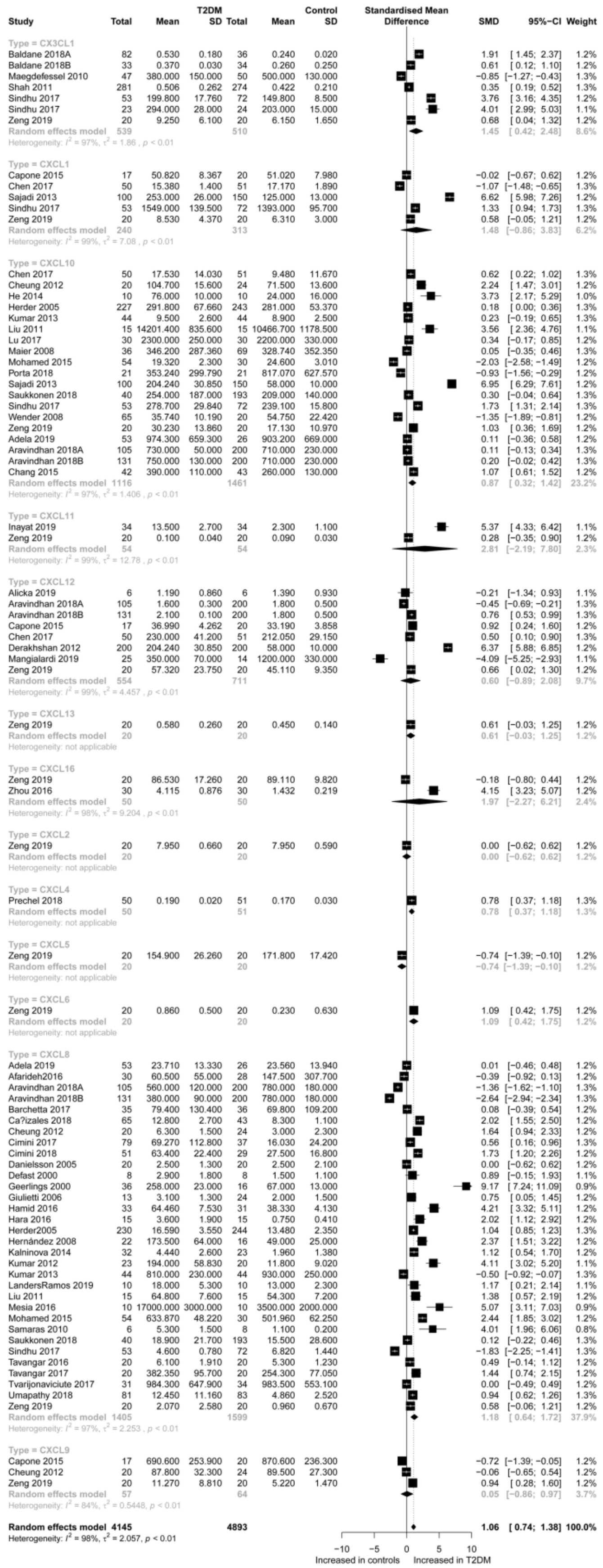

Study effect sizes of CXC chemokine differences between T2DM and controls. Each data marker represents a study, and the size of the data marker is proportional to the total number of individuals in that study. The summary effect size for each CXC chemokine is denoted by a diamond. T2DM, Type-2 diabetes mellitus; SMD, standardized mean difference.

Appendix 6: Forest plot of chemokine between PDM patients and controls

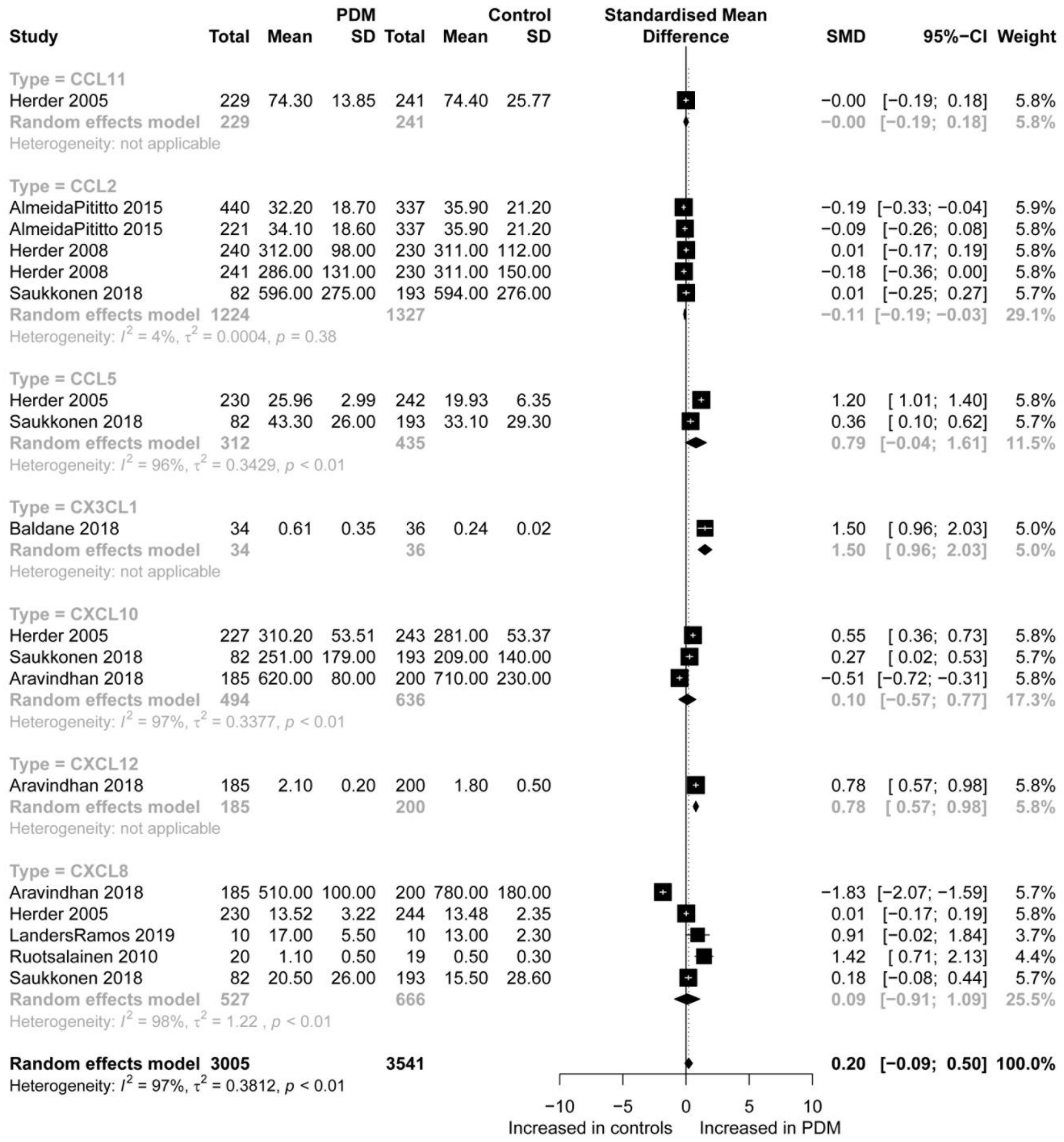

Study effect sizes of chemokine differences between PDM and controls. Each data marker represents a study, and the size of the data marker is proportional to the total number of individuals in that study. The summary effect size for each chemokine is denoted by a diamond. PDM, prediabetes mellitus; SMD, standardized mean difference.

Appendix 7: Egger funnel plots of T2DM patients compared to controls.

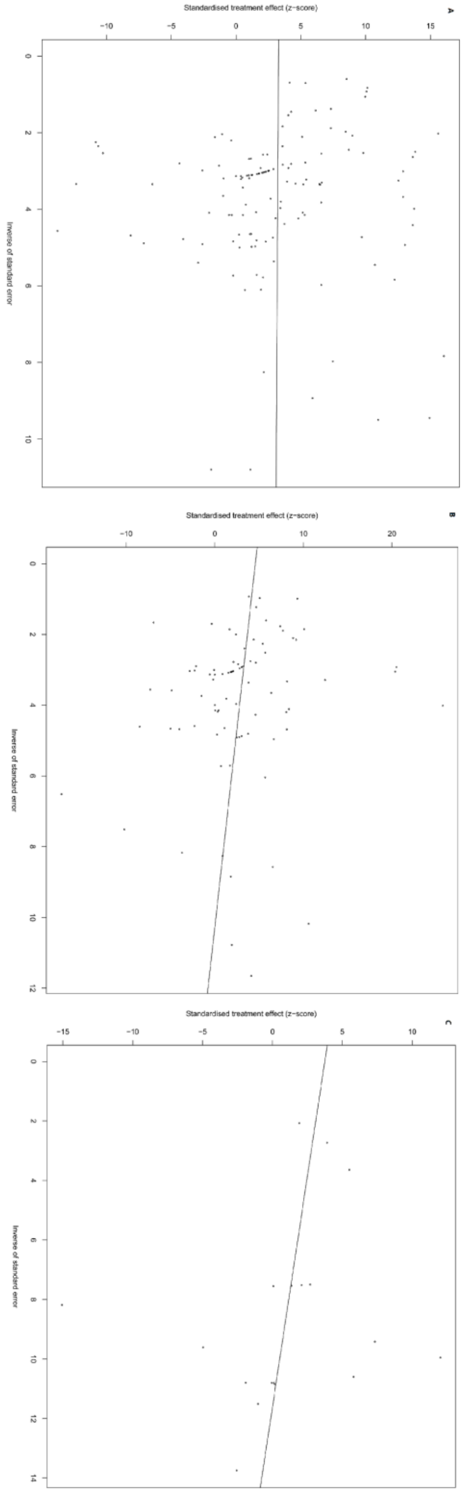

T2DM patients CC chemokine compared to patients with controls (A), t = 1.5321, p-value = 0.1368, T2DM patients CXC chemokine compared to controls (B), t = 1.2385, p-value = 0.2543, PDM patients chemokine compared to controls (C), t = 0.92878, p-value = 0.3668. Egger funnel plots to assess publication bias. Plots show study size as a function of effect size for studies included in the meta-analysis. The dots represent each study

Appendices References

1 Adela R, Reddy PNC, Ghosh TS, Aggarwal S, Yadav AK, Das B, et al. Serum protein signature of coronary artery disease in type 2 diabetes mellitus. J Transl Med. 2019;17(1):17.

2 Afarideh M, Ghanbari P, Noshad S, Ghajar A, Nakhjavani M, Esteghamati A. Raised serum 25-hydroxyvitamin D levels in patients with active diabetic foot ulcers. Br J Nutr. 2016;115(11):1938-46.

3 Ahmed SF, Shabayek MI, Ghany MEA, El-Hefnawy MH, El-Mesallamy HO. Role of CTRP3, CTRP9 and MCP-1 for the evaluation of T2DM associated coronary artery disease in Egyptian postmenopausal females. Plos One. 2018;13(12).

4 Alicka M, Major P, Wysocki M, Marycz K. Adipose-Derived Mesenchymal Stem Cells Isolated from Patients with Type 2 Diabetes Show Reduced "Stemness" through an Altered Secretome Profile, Impaired Anti-Oxidative Protection, and Mitochondrial Dynamics Deterioration. Journal of clinical medicine. 2019;8(6).

5 Almeida-Pititto B, Ribeiro-Filho FF, Lotufo PA, Bensenor IM, Ferreira SR. Novel biomarkers of cardiometabolic risk are associated with plasma glucose within non-diabetic range. The Brazilian Longitudinal Study of Adult Health - ELSA-Brasil. Diabetes Res Clin Pract. 2015;109(1):110-6.

6 Alvarado-Vazquez PA, Grosick RL, Moracho-Vilrriales C, Ward E, Threatt T, Romero-Sandoval EA. Cytokine production capabilities of human primary monocyte-derived macrophages from patients with diabetes mellitus type 2 with and without diabetic peripheral neuropathy. J Pain Res. 2019;12:69-81.

7 Aravindhan V, Kevinkumar V, Dhamodharan U, Viswanathan V. Serum levels of chemokines IP-10, IL-8 and SDF-1 serve as good biomarkers for diabetes-tuberculosis nexus. Journal of diabetes and its complications. 2018;32(9):857-62.

8 Bala M, Kopp A, Wurm S, Büchler C, Schölmerich J, Schäffler A. Type 2 diabetes and lipoprotein metabolism affect LPS-induced cytokine and chemokine release in primary human monocytes. Experimental and Clinical Endocrinology and Diabetes. 2011;119(6):370-76.

9 Baldane S, Kendir IC, Kirac CO, Ipekci S, Tekin G, Unlu A, et al. EFFECTS OF GLUCOSE INGESTION ON SERUM FRACTALKINE LEVELS IN HEALTHY SUBJECTS AND NEWLY DIAGNOSED TYPE 2 DIABETIC PATIENTS. Journal of Medical Biochemistry. 2018;37(3):373-78.

10 Baldane S, Ipekci SH, Ekin A, Abusoglu S, Unlu A, Kebapcilar L. Evaluation of fractalkine (FKN) and secreted frizzled-related protein 4 (SFRP-4) serum levels in patients with prediabetes and type 2 diabetes. Bratislava Medical Journal-Bratislavske Lekarske Listy. 2018;119(2):112-15.

11 Barchetta I, Cimini FA, Capoccia D, De Gioannis R, Porzia A, Mainiero F, et al. WISP1 Is a Marker of Systemic and Adipose Tissue Inflammation in Dysmetabolic Subjects With or Without Type 2 Diabetes. Journal of the Endocrine Society. 2017;1(6):660-70.

12 Cataño Cañizales YG, Uresti Rivera EE, García Jacobo RE, Portales Perez DP, Yadira B, Rodriguez Rivera JG, et al. Increased Levels of AIM2 and Circulating Mitochondrial DNA in Type 2 Diabetes. Iranian journal of immunology : IJI. 2018;15(2):142-55.

13 Capone F, Guerriero E, Colonna G, Maio P, Mangia A, Marfella R, et al. The Cytokinome Profile in Patients with Hepatocellular Carcinoma and Type 2 Diabetes. PLoS One. 2015;10(7):e0134594.

14 Cha JJ, Hyun YY, Jee YH, Lee MJ, Han KH, Kang YS, et al. Plasma leptin concentrations are greater in type II diabetic patients and stimulate monocyte chemotactic peptide-1 synthesis via the mitogen-activated protein kinase/extracellular signal-regulated kinase pathway. Kidney research and clinical practice. 2012;31(3):177-85.

15 Chang C-C, Wu C-L, Su W-W, Shih K-L, Tarng D-C, Chou C-T, et al. Interferon gamma-induced protein 10 is associated with insulin resistance and incident diabetes in patients with nonalcoholic fatty liver disease. Scientific reports. 2015;5.

16 Chao PC, Huang CN, Hsu CC, Yin MC, Guo YR. Association of dietary AGEs with circulating AGEs, glycated LDL, IL-1α and MCP-1 levels in type 2 diabetic patients. European Journal of Nutrition. 2010;49(7):429-34.

17 Chen H, Zhang X, Liao N, Wen F. Assessment of biomarkers using multiplex assays in aqueous humor of patients with diabetic retinopathy. BMC ophthalmology. 2017;17(1):176.

18 Cheng M, Li BY, Li XL, Wang Q, Zhang JH, Jing XJ, et al. Correlation between serum lactadherin and pulse wave velocity

and cardiovascular risk factors in elderly patients with type 2 diabetes mellitus. Diabetes Res Clin Pract. 2012;95(1):125-31.

19 Cheung CMG, Vania M, Ang M, Chee SP, Li J. Comparison of aqueous humor cytokine and chemokine levels in diabetic patients with and without retinopathy. Molecular Vision. 2012;18(87-88):830-37.

20 Cimini FA, Barchetta I, Porzia A, Mainiero F, Costantino C, Bertoccini L, et al. Circulating IL-8 levels are increased in patients with type 2 diabetes and associated with worse inflammatory and cardiometabolic profile. Acta Diabetologica. 2017;54(10):961-67.

21 Cimini FA, D'Eliseo D, Barchetta I, Bertoccini L, Velotti F, Cavallo MG. Increased circulating granzyme B in type 2 diabetes patients with low-grade systemic inflammation. Cytokine. 2019;115:104-08.

22 Danielsson P, Truedsson L, Eriksson KF, Norgren L. Inflammatory markers and IL-6 polymorphism in peripheral arterial disease with and without diabetes mellitus. Vascular medicine (London, England). 2005;10(3):191-8.

23 Davì G, Tuttolomondo A, Santilli F, Basili S, Ferrante E, Di Raimondo D, et al. CD40 ligand and MCP-1 as predictors of cardiovascular events in diabetic patients with stroke. Journal of atherosclerosis and thrombosis. 2009;16(6):707-13.

24 Desfaits AC, Serri O, Renier G. Normalization of plasma lipid peroxides, monocyte adhesion, and tumor necrosis factor-alpha production in NIDDM patients after gliclazide treatment. Diabetes Care. 1998;21(4):487-93.

25 Degirmenci I, Ozbayer C, Kebapci MN, Kurt H, Colak E, Gunes HV. Common variants of genes encoding TLR4 and TLR4 pathway members TIRAP and IRAK1 are effective on MCP1, IL6, IL1beta, and TNFalpha levels in type 2 diabetes and insulin resistance. Inflammation research : official journal of the European Histamine Research Society [et al]. 2019;68(9):801-14.

26 Derakhshan R, Arababadi MK, Ahmadi Z, Karimabad MN, Salehabadi VA, Abedinzadeh M, et al. Increased circulating levels of SDF-1 (CXCL12) in type 2 diabetic patients are correlated to disease state but are unrelated to polymorphism of the SDF-1β gene in the Iranian population. Inflammation. 2012;35(3):900-04.

27 El-Mesallamy HO, Hamdy NM, Salman TM, Ibrahim SM. Adiponectin and sE-selectin concentrations in relation to inflammation in obese type 2 diabetic patients with coronary heart disease. Angiology. 2012;63(2):96-102.

28 Feng S, Yu H, Yu Y, Geng Y, Li D, Yang C, et al. Levels of inflammatory cytokines IL-1β, IL-6, IL-8, IL-17A, and TNF-α in aqueous humour of patients with diabetic retinopathy. Journal of diabetes research. 2018;2018.

29 Funatsu H, Noma H, Mimura T, Eguchi S, Hori S. Association of vitreous inflammatory factors with diabetic macular edema. Ophthalmology. 2009;116(1):73-9.

30 Geerlings SE, Brouwer EC, Van Kessel KC, Gaastra W, Stolk RP, Hoepelman AI. Cytokine secretion is impaired in women with diabetes mellitus. Eur J Clin Invest. 2000;30(11):995-1001.

31 Giulietti A, van Etten E, Overbergh L, Stoffels K, Bouillon R, Mathieu C. Monocytes from type 2 diabetic patients have a pro-inflammatory profile. 1,25-Dihydroxyvitamin D(3) works as anti-inflammatory. Diabetes Res Clin Pract. 2007;77(1):47-57.

32 Gokulakrishnan K, Amutha A, Ranjani H, Bibin SY, Balakumar M, Pandey GK, et al. Relationship of Adipokines and Proinflammatory Cytokines among Asian Indians with Obesity and Youth Onset Type 2 Diabetes. Endocrine practice : official journal of the American College of Endocrinology and the American Association of Clinical Endocrinologists. 2015;21(10):1143-51.

33 Gomez JM, Vila R, Catalina P, Soler J, Badimon L, Sahun M. The markers of inflammation and endothelial dysfunction in correlation with glycated haemoglobin are present in type 2 diabetes mellitus patients but not in their relatives. Glycoconjugate journal. 2008;25(6):573-9.

34 Gong F, Wu J, Zhou P, Zhang M, Liu J, Liu Y, et al. Interleukin-22 Might Act as a Double-Edged Sword in Type 2 Diabetes and Coronary Artery Disease. Mediators Inflamm. 2016;2016:8254797.

35 Hamid S, Gul A, Hamid Q. Relationship of cytokines and AGE products in diabetic and non-diabetic patients with cataract. International journal of health sciences. 2016;10(4):507-15.

36 Hara Cde C, Franca EL, Fagundes DL, de Queiroz AA, Rudge MV, Honorio-Franca AC, et al. Characterization of Natural Killer Cells and Cytokines in Maternal Placenta and Fetus of Diabetic Mothers. Journal of immunology research. 2016;2016:7154524.

37 He L, Wong CK, Cheung KK, Yau HC, Fu A, Zhao HL, et al. Anti-inflammatory effects of exendin-4, a glucagon-like peptide-1 analog, on human peripheral lymphocytes in patients with type 2 diabetes. J Diabetes Investig. 2013;4(4):382-92.

38 Herder C, Müller-Scholze S, Rating P, Koenig W, Thorand B, Haastert B, et al. Systemic monocyte chemoattractant protein-1 concentrations are independent of type 2 diabetes or parameters of obesity: results from the Cooperative Health Research in the Region of Augsburg Survey S4 (KORA S4). Eur J Endocrinol. 2006;154(2):311-7.

39 Herder C, Illig T, Baumert J, Müller M, Klopp N, Khuseyinova N, et al. RANTES/CCL5 gene polymorphisms, serum concentrations, and incident type diabetes: Results from the MONICA/KORA Augsburg case-cohort study, 1984-2002. European Journal of Endocrinology. 2008;158(5):R1-R5.

40 Hernández C, Segura RM, Fonollosa A, Carrasco E, Francisco G, Simó R. Interleukin-8, monocyte chemoattractant protein-1 and IL-10 in the vitreous fluid of patients with proliferative diabetic retinopathy. Diabet Med. 2005;22(6):719-22.

41 Hirsch FF, Pareja JC, Geloneze SR, Chaim E, Cazzo E, Geloneze B. Comparison of metabolic effects of surgical-induced massive weight loss in patients with long-term remission versus non-remission of type 2 diabetes. Obes Surg. 2012;22(6):910-7.

42 Hu YY, Ye SD, Zhao LL, Zheng M, Wu FZ, Chen Y. Hydrochloride pioglitazone decreases urinary cytokines excretion in type 2 diabetes. Clinical endocrinology. 2010;73(6):739-43.

43 Huang G, Mo X, Li M, Xiang Y, Li X, Luo S, et al. Autoantibodies to CCL3 are of low sensitivity and specificity for the diagnosis of type 1 diabetes. Acta Diabetologica. 2012;49(5):395-99.

44 Inayat H, Azim MK, Baloch AA. Analysis of Inflammatory Gene Expression Profile of Peripheral Blood Leukocytes in Type 2 Diabetes. Immunological investigations. 2019;48(6):618-31.

45 Kalninova J, Jakus V, Glejtkova M, Kuracka L, Sandorova E. Impact of glycemic control on advanced glycation and inflammation in overweight and obese patients with type 2 diabetes mellitus. Bratislavske lekarske listy. 2014;115(8):457-68.

46 Kang YS, Song HK, Lee MH, Ko GJ, Cha DR. Plasma concentration of visfatin is a new surrogate marker of systemic inflammation in type 2 diabetic patients. Diabetes Res Clin Pract. 2010;89(2):141-9.

47 Kou H, Deng J, Gao D, Song A, Han Z, Wei J, et al. Relationship among adiponectin, insulin resistance and atherosclerosis in non-diabetic hypertensive patients and healthy adults. Clinical and experimental hypertension (New York, NY : 1993). 2018;40(7):656-63.

48 Pavan Kumar P, Radhika G, Rao GV, Pradeep R, Subramanyam C, Talukdar R, et al. Interferon γ and glycemic status in diabetes associated with chronic pancreatitis. Pancreatology : official journal of the International Association of Pancreatology (IAP) [et al]. 2012;12(1):65-70.

49 Kumar NP, Sridhar R, Banurekha VV, Jawahar MS, Fay MP, Nutman TB, et al. Type 2 diabetes mellitus coincident with pulmonary tuberculosis is associated with heightened systemic type 1, type 17, and other proinflammatory cytokines. Annals of the American Thoracic Society. 2013;10(5):441-9.

50 Landers-Ramos RQ, Blumenthal JB, Prior SJ. Serum IL-6 and sIL-6R in type 2 diabetes contribute to impaired capillary-like network formation. J Appl Physiol (1985). 2019;127(2):385-92.

51 Lareyre F, Moratal C, Chikande J, Jean-Baptiste E, Hassen-Khodja R, Neels J, et al. Investigation of Plasma Inflammatory Profile in Diabetic Patients With Abdominal Aortic Aneurysm: A Pilot Study. Vascular and endovascular surgery. 2018;52(8):597-601.

52 Li C, Kang D, Sun X, Liu Y, Wang J, Gao P. The Effect of C-X-C Motif Chemokine 13 on Hepatocellular Carcinoma Associates with Wnt Signaling. Biomed Res Int. 2015;2015:345413.

53 Liu S, Huang J, Yu F, Wang J, Zhang Q, Qu S, et al. The expression of chemerin and chemerin receptor in MCD induced NAFLD rat. Diabetes. 2011;60:A686.

54 Liu T, Xue R, Dong L, Wu H, Zhang D, Shen X. Rapid determination of serological cytokine biomarkers for hepatitis B virus-related hepatocellular carcinoma using antibody microarrays. Acta Biochim Biophys Sin (Shanghai). 2011;43(1):45-51.

55 Liuni FM, Rugiero C, Feola M, Rao C, Pistillo P, Terracciano C, et al. Impaired healing of fragility fractures in type 2 diabetes: clinical and radiographic assessments and serum cytokine levels. Aging clinical and experimental research. 2015;27 Suppl 1:S37-44.

56 Lu P, Ji X, Wan J, Xu H. Activity of Group 2 Innate Lymphoid Cells is Associated with Chronic Inflammation and Dysregulated Metabolic Homoeostasis in Type 2 Diabetic Nephropathy. Scand J Immunol. 2018;87(2):99-107.

57 Maegdefessel L, Schlitt A, Pippig S, Schwaab B, Fingscheidt K, Raaz U, et al. Patients with insulin-dependent diabetes or coronary heart disease following rehabilitation express serum fractalkine levels similar to those in healthy control subjects. Vascular health and risk management. 2009;5:849-57.

58 Maier R, Weger M, Haller-Schober EM, El-Shabrawi Y, Wedrich A, Theisl A, et al. Multiplex bead analysis of vitreous and serum concentrations of inflammatory and proangiogenic factors in diabetic patients. Mol Vis. 2008;14:637-43.

59 Mangialardi G, Ferland-McCollough D, Maselli D, Santopaolo M, Cordaro A, Spinetti G, et al. Bone marrow pericyte dysfunction in individuals with type 2 diabetes. Diabetologia. 2019;62(7):1275-90.

60 McCarthy CP, Shrestha S, Ibrahim N, van Kimmenade RRJ, Gaggin HK, Mukai R, et al. Performance of a clinical/proteomic panel to predict obstructive peripheral artery disease in patients with and without diabetes mellitus. Open heart. 2019;6(1):e000955.

61 Mesia R, Gholami F, Huang H, Clare-Salzler M, Aukhil I, Wallet SM, et al. Systemic inflammatory responses in patients with type 2 diabetes with chronic periodontitis. BMJ open diabetes research & care. 2016;4(1):e000260.

62 Mine S, Okada Y, Tanikawa T, Kawahara C, Tabata T, Tanaka Y. Increased expression levels of monocyte CCR2 and monocyte chemoattractant protein-1 in patients with diabetes mellitus. Biochemical and Biophysical Research Communications. 2006;344(3):780-85.

63 Mohamed HG, Idris SB, Ahmed MF, Astrom AN, Mustafa K, Ibrahim SO, et al. Influence of type 2 diabetes on local production of inflammatory molecules in adults with and without chronic periodontitis: a cross-sectional study. BMC oral health. 2015;15:86.

64 Murase H, Suzuki E, Tajima Y, Hayashi K, Nakamura T, Noritake N, et al. Associations of plasma von Willebrand factor ristocetin cofactor activity and 5-hydroxyindole acetic acid concentrations with blood flow in lower-leg arteries in Japanese type 2 diabetic patients with normal ankle-brachial index. Journal of diabetes and its complications. 2012;26(2):113-7.

65 Nomura S, Shouzu A, Omoto S, Nishikawa M, Iwasaka T. Benidipine improves oxidized LDL-dependent monocyte and endothelial dysfunction in hypertensive patients with type 2 diabetes mellitus. Journal of human hypertension. 2005;19(7):551-7.

66 Omoto S, Taniura T, Nishizawa T, Tamaki T, Shouzu A, Nomura S. Anti-atherosclerotic effects of sitagliptin in patients with type 2 diabetes mellitus. Diabetes, metabolic syndrome and obesity : targets and therapy. 2015;8:339-45.

67 Papatheodorou K, Papanas N, Papazoglou D, Gioka T, Antonoglou C, Glaros D, et al. Monocyte chemoattractant protein 1 is correlated with glycemic control and peripheral arterial disease in type 2 diabetic patients with metabolic syndrome. Angiology. 2013;64(3):223-9.

68 Pham MN, Hawa MI, Roden M, Schernthaner G, Pozzilli P, Buzzetti R, et al. Increased serum concentrations of adhesion molecules but not of chemokines in patients with Type2 diabetes compared with patients with Type1 diabetes and latent autoimmune diabetes in adult age: Action LADA5. Diabetic Medicine. 2012;29(4):470-78.

69 Porta M, Amione C, Barutta F, Fornengo P, Merlo S, Gruden G, et al. The co-activator-associated arginine methyltransferase 1 (CARM1) gene is overexpressed in type 2 diabetes. Endocrine. 2019;63(2):284-92.

70 Prechel M, Hudec S, Lowden E, Escalante V, Emanuele N, Emanuele M, et al. Profiling Heparin-Induced Thrombocytopenia (HIT) Antibodies in Hospitalized Patients With and Without Diabetes. Clinical and applied thrombosis/hemostasis : official journal of the International Academy of Clinical and Applied Thrombosis/Hemostasis. 2018;24(9_suppl):294S-300S.

71 Pushpanathan P, Srikanth P, Seshadri KG, Selvarajan S, Pitani RS, Kumar TD, et al. Gut Microbiota in Type 2 Diabetes Individuals and Correlation with Monocyte Chemoattractant Protein1 and Interferon Gamma from Patients Attending a Tertiary Care Centre in Chennai, India. Indian J Endocrinol Metab. 2016;20(4):523-30.

72 Ruotsalainen E, Stancakova A, Vauhkonen I, Salmenniemi U, Pihlajamaki J, Punnonen K, et al. Changes in cytokine levels during acute hyperinsulinemia in offspring of type 2 diabetic subjects. Atherosclerosis. 2010;210(2):536-41.

73 Sajadi SMA, Khoramdelazad H, Hassanshahi G, Rafatpanah H, Hosseini J, Mahmoodi M, et al. Plasma levels of CXCL1 (GRO-α) and CXCL10 (IP-10) are elevated in type 2 diabetic patients: Evidence for the involvement of inflammation and angiogenesis / angiostasis in this disease state. Clinical Laboratory. 2013;59(1-2):133-37.

74 Samaras K, Botelho NK, Chisholm DJ, Lord RV. Subcutaneous and visceral adipose tissue gene expression of serum adipokines that predict type 2 diabetes. Obesity (Silver Spring, Md). 2010;18(5):884-9.

75 Sathishkumar C, Prabu P, Balakumar M, Lenin R, Prabhu D, Anjana RM, et al. Augmentation of histone deacetylase 3 (HDAC3) epigenetic signature at the interface of proinflammation and insulin resistance in patients with type 2 diabetes. Clinical epigenetics. 2016;8:125.

76 Saukkonen T, Mutt SJ, Jokelainen J, Saukkonen AM, Raza GS, Karhu T, et al. Adipokines and inflammatory markers in elderly subjects with high risk of type 2 diabetes and cardiovascular disease. Scientific reports. 2018;8(1):12816.

77 Shah R, Hinkle CC, Ferguson JF, Mehta NN, Li M, Qu L, et al. Fractalkine is a novel human adipochemokine associated with type 2 diabetes. Diabetes. 2011;60(5):1512-18.

78 Sindhu S, Akhter N, Shenouda S, Wilson A, Ahmad R. Plasma fetuin-A/alpha 2-HS-glycoprotein correlates negatively with inflammatory cytokines, chemokines and activation biomarkers in individuals with type-2 diabetes. Bmc Immunology. 2016;17.

79 Sindhu S, Akhter N, Arefanian H, Al-Roub AA, Ali S, Wilson A, et al. Increased circulatory levels of fractalkine (CX3CL1) are associated with inflammatory chemokines and cytokines in individuals with type-2 diabetes. Journal of diabetes and metabolic disorders. 2017;16(1).

80 Sozer V, Himmetoglu S, Korkmaz GG, Kaya S, Aydin S, Yumuk V, et al. Paraoxonase, oxidized low density lipoprotein, monocyte chemoattractant protein-1 and adhesion molecules are associated with macrovascular complications in patients with type 2 diabetes mellitus. Minerva Med. 2014;105(3):237-44.

81 Tavangar A, Khozeimeh F, Ghoreishian F, Boroujeni MA. Serum level of Interleukin-8 in subjects with diabetes, diabetes plus oral lichen planus, and oral lichen planus: A biochemical study. Dental research journal. 2016;13(5):413-18.

82 Tavangar A, Ghalayani P, Boroujeni MA, Ghoreishian FS. Salivary levels of interleukin-8 in oral lichen planus and diabetic patients: A biochemical study. Dental research journal. 2017;14(3):209-14.

83 Toan NL, Van Hoan N, Cuong DV, Dung NV, Dung PT, Hang NT, et al. Adipose tissue-derived cytokines and their correlations with clinical characteristics in Vietnamese patients with type 2 diabetes mellitus. Diabetol Metab Syndr. 2018;10:41.

84 Tokarz A, Drozdz AE, Szuzcik I, Stzpiez E. Interplay of RANTES chemokine and CCR5+ bearing microvesicles in diabetic retinopathy. Journal of Extracellular Vesicles. 2017;6:119.

85 Tvarijonaviciute A, Castillo C, Ceron JJ, Martinez-Subiela S, Tecles F, Lopez-Jornet P. Leptin and NGF in saliva of patients with diabetes mellitus type 2: A pilot study. Journal of oral pathology & medicine : official publication of the International Association of Oral Pathologists and the American Academy of Oral Pathology. 2017;46(9):853-55.

86 Umapathy D, Dornadula S, Krishnamoorthy E, Mariappanadar V, Viswanathan V, Ramkumar KM. YKL-40: A biomarker for early nephropathy in type 2 diabetic patients and its association with inflammatory cytokines. Immunobiology. 2018;223(11):718-27.

87 Wada T, Furuichi K, Sakai N, Iwata Y, Yoshimoto K, Shimizu M, et al. Up-regulation of monocyte chemoattractant protein-1 in tubulointerstitial lesions of human diabetic nephropathy. Kidney Int. 2000;58(4):1492-9.

88 Wang X, Li J, Wang Z, Deng A. Wound exudate CXCL6: a potential biomarker for wound healing of diabetic foot ulcers. Biomarkers in Medicine. 2019;13(3):167-74.

89 Wei J, Tang Q, Liu L, Bin J. Combination of peroxisome proliferator-activated receptor alpha/gamma agonists may benefit type 2 diabetes patients with coronary artery disease through inhibition of inflammatory cytokine secretion. Exp Ther Med. 2013;5(3):783-88.

90 Wender-Ozegowska E, Michałowska-Wender G, Zawiejska A, Pietryga M, Brazert J, Wender M. Concentration of chemokines in peripheral blood in first trimester of diabetic pregnancy. Acta Obstetricia et Gynecologica Scandinavica.

2008;87(1):14-19.
91 Wu C, Wang Q, Lv C, Qin N, Lei S, Yuan Q, et al. The changes of serum sKlotho and NGAL levels and their correlation in type 2 diabetes mellitus patients with different stages of urinary albumin. Diabetes Res Clin Pract. 2014;106(2):343-50.
92 Xu J, Liao YF, Zhou WP, Ming HL, Wang QH. The MCP-1 Gene A-2518G Polymorphism Confers an Increased Risk of Vascular Complications in Type 2 Diabetes Mellitus Patients. Genetic testing and molecular biomarkers. 2015;19(8):411-17.
93 Yadav R, Hama S, Liu Y, Siahmansur T, Schofield J, Syed AA, et al. Effect of Roux-en-Y Bariatric Surgery on Lipoproteins, Insulin Resistance, and Systemic and Vascular Inflammation in Obesity and Diabetes. Frontiers in immunology. 2017;8:1512.
94 Yang M, Shen Z, Chen D, Gan H, Shen Q, Yang B, et al. Effects of 1,25-(OH)(2)D (3) on the expressions of vitamin D receptor, STAT5 and cytoskeletal rearrangement in human monocytes incubated with sera from type 2 diabetes patients and diabetic nephropathy patients with uremia. Inflammation research : official journal of the European Histamine Research Society [et al]. 2012;61(5):511-20.
95 Yi B, Hu X, Zhang H, Huang J, Liu J, Hu J, et al. Nuclear NF-kappaB p65 in peripheral blood mononuclear cells correlates with urinary MCP-1, RANTES and the severity of type 2 diabetic nephropathy. PLoS One. 2014;9(6):e99633.
96 Zeng Y, Cao D, Yu H, Hu Y, He M, Yang D, et al. Comprehensive analysis of vitreous humor chemokines in type 2 diabetic patients with and without diabetic retinopathy. Acta Diabetologica. 2019;56(7):797-805.
97 Zhang J, Zhang Z, Ding Y, Xu P, Wang T, Xu W, et al. Adipose Tissues Characteristics of Normal, Obesity, and Type 2 Diabetes in Uygurs Population. Journal of diabetes research. 2015;2015:905042.
98 Zhou F, Wang J, Wang K, Zhu X, Pang R, Li X, et al. Serum CXCL16 as a Novel Biomarker of Coronary Artery Disease in Type 2 Diabetes Mellitus: a Pilot Study. Annals of Clinical and Laboratory Science. 2016;46(2):184-89.
